# The Spicy Science of Dendrimers in the Realm of Cancer Nanomedicine: A Report from the COST Action CA17140 Nano2Clinic

**DOI:** 10.3390/pharmaceutics15072013

**Published:** 2023-07-24

**Authors:** Sabrina Pricl

**Affiliations:** 1 Molecular Biology and Nanotechnology Laboratory (MolBNL@UniTS), Department of Engineering and Architecture (DEA), University of Trieste, Piazzale Europa 1, 34127 Trieste, Italy; sabrina.pricl@dia.units.it; Tel.: +39-0405583750; 2 Department of General Biophysics, Faculty of Biology and Environmental Protection, University of Lodz, ul. Pomorska 141/143, 90-236 Lodz, Poland

**Keywords:** dendrimers, dendrons, self-assembly, dendrimersomes, cancer nanomedicine, nanotheranostics, dendrimer design, dendrimer characterization, in vitro activity, in vivo activity

## Abstract

COST Action CA17140 Cancer Nanomedicine—from the bench to the bedside (Nano2Clinic,) is the first, pan-European interdisciplinary network of representatives from academic institutions and small and medium enterprises including clinical research organizations (CROs) devoted to the development of nanosystems carrying anticancer drugs from their initial design, preclinical testing of efficacy, pharmacokinetics and toxicity to the preparation of detailed protocols needed for the first phase of their clinical studies. By promoting scientific exchanges, technological implementation, and innovative solutions, the action aims at providing a timely instrument to rationalize and focus research efforts at the European level in dealing with the grand challenge of nanomedicine translation in cancer, one of the major and societal-burdening human pathologies. Within CA17140, dendrimers in all their forms (from covalent to self-assembling dendrons) play a vital role as powerful nanotheranostic agents in oncology; therefore, the purpose of this review work is to gather and summarize the major results in the field stemming from collaborative efforts in the framework of the European Nano2Clinic COST Action.

## 1. The COST Action CA17140 Cancer Nanomedicine—From the Bench to the Bedside and Dendrimers: A Natural Liaison

Finding efficient cancer therapies is an urgent and still unresolved problem, and, in the fight against this disease, scientists are devoting tremendous efforts to harness the multi-faceted power of cancer nanotechnology [1,2,3,4,5,6]. Nanotherapeutics exhibit major benefits over unmodified drugs, including—among others—improved half-life, more efficient tumor targeting, and reduced side effects [7,8,9,10]. However, only a few nanotherapeutics have reached the commercial level, most of them still being in the investigational phase [11,12,13,14,15,16,17,18]. Consequently, the ongoing European Cooperation in Science and Technology (COST) Action CA17140 Cancer Nanomedicine—from the bench to the bedside (https://www.nano2clinic.eu/) aims at developing and strengthening industry-academia relations with an ultimate goal: fostering the clinical translation of cancer nanomedicine. To this end, CA17140 has assembled the first pan-European interdisciplinary network of representatives from academic institutions, small and medium enterprises and clinical research organizations exquisitely focused on the entire pipeline of anticancer nanomedicines, i.e., from their initial design, pre-clinical testing of efficacy, pharmacokinetics, and toxicity to the preparation of detailed protocols needed for the first phase of their clinical studies. By promoting scientific exchanges, technological implementation and innovative solutions, CA17140 currently provides a timely instrument to rationalize and focus research efforts at the European Union (EU) level in dealing with the grand challenge of nanomedicine translation in cancer, one of the major and societal burdening human pathologies. The activities within CA17140 are organized into four major working groups (WG1), namely:

- WG1—“Manufacturing nanodrugs”, in which computational scientists concur in the design of new chemical entities, the optimization of existing chemical structures and the formulation of new nanomaterials in silico while chemists in different EU laboratories synergize in producing relevant synthetic protocols according to Good Manufacturing Practice (GMP);
- WG2—“Physicochemical characterization of nanodrugs”, in which the main tasks and activities consist of performing a complete physicochemical characterization and quality control of all nanomedicines produced in WG1 by using all available state-of-the-art techniques;
- WG3—“Preclinical studies of nanodrugs”, where biologists and pharmacologists gather all the information necessary to demonstrate a complete understanding of the mechanism of action of new nanomedicines, along with their most relevant properties and activity in vitro and in vivo;
- WG4—“Guidelines for clinical trials and regulatory aspects of nanomedicines”, a working group devoted to the necessary steps to foster the translation of the developed nanomedicine cancer drug to bed/market (e.g., preparation of guidelines describing in detail the objectives, protocol design, methodology, statistical considerations, and organization of eventual early clinical trials for the developed nanomedicines).

Although dendrimers in all their forms (from covalent molecules to self-assembling dendrons) appeared on the horizon only a few decades ago, they soon showed significant potential as drug/gene delivery and theranostic nanovectors in pharmaceutical research, innovation, and other biomedical/healthcare applications. Accordingly, these molecules quickly catalyzed the attention and the activity of the scientific community, resulting in a large number of medicine-related articles and patents (for a selection of very recent reviews on the subject, see [19,20,21] and references therein). This, in turn, generated hypes and hopes for their successful clinical translation, particularly in the field of personalized medicine. Rather disappointingly, however, all these major efforts have resulted in a relatively limited number of clinical trials involving dendrimers to date [19,22]. Indeed, even though dendrimer chemistry and the associated biomedical studies and applications have greatly progressed since their inception, there are still substantial challenges to be faced before these nanostructures can definitely and successfully enter the clinics. And contributing to removing such obstacles is exactly the spirit of the COST Action CA17140, where dendrimers and dendrons (as such, in their different self-assembled form or as decorating moieties of, e.g., inorganic nanoparticles) play a major role as prospective powerful theranostic agents in nanooncology. Accordingly, the purpose of this review is to briefly report on the collaborative work performed by WG1–WG3 in the framework of the CA17140 initiative along the long and winding road of moving these fascinating compounds from the bench to the bedside in cancer nanomedicine. The paper is organized into two major sections (Section 2 and Section 3), the first focusing on covalent dendrimers and the second dealing with self-assembling dendrimers and dendrons. Section 2 is further organized into four subsections, discussing the interaction of covalent dendrimers with biological membranes and human serum albumin (2.1), the role of covalent dendrimers in small drug delivery (2.2), the use of covalent dendrimers as anticancer agents per se (2.3), and the delivery of nucleic acids by these covalent hyperbranched molecules (2.4). Section 3 begins with a part dedicated to self-assembling dendrimers in drug delivery (3.1), followed by another, reporting on their nucleic acid delivery (3.2). Section 4 and Section 5 deal with two particular topics, that is, dendrimer-decorated metal nanoparticles as anticancer delivery agents and self-assembling dendrimers in cancer bioimaging, respectively. Finally, Section 6 collects the work performed within CA17140 on various ensembles of dendrimer-based molecules and nanoparticles for applications beyond cancer.

## 2. Covalent Dendrimers

### 2.1. Covalent Dendrimers and Their Interactions with Biological Membranes and Human Serum Albumin

**Figure 1:**
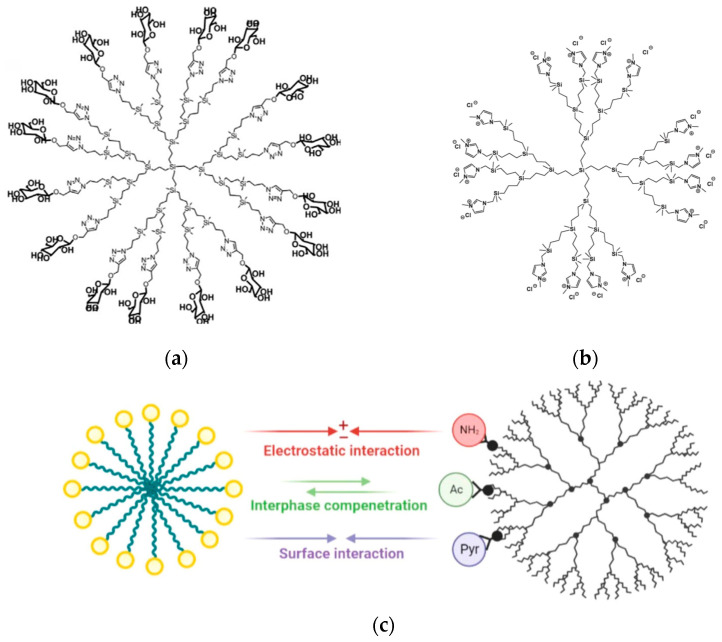
(**a**) Molecular structure of the G3 glucose-modified carbosilane dendrimer (**DDM_3_Glu**). Adapted with permission from [25]. Copyright 2020, Elsevier. (**b**) Model structure of the G3 imidazolium-terminated carbosilane dendrimer. Adapted with permission from [26]. Copyright 2020, Elsevier. (**c**) Outline of the distinct patterns of interaction exhibited by PAMAM-based dendrimers with different surface terminations (amine, -HN_2_; acetamide, Ac; and 3-methoxy-carbonyl-5-pyrrolidonyl, Pyr), with SDS micelles. Adapted with permission from [27]. Copyright 2022, American Chemical Society.

Carbosilane dendrimers (CSDs) are a class of hyperbranched molecules widely explored for a number of potential biomedical applications, including drug/nucleic acid delivery systems in anticancer/anti-HIV treatment and as nanomaterials endowed with biological activity per se [23,24]. In this field, a joint Polish–Czech group investigated the interactions between glucose-modified CSDs (first to third generation **DDM_1–3_Glus**, Figure 1a) and two biological models: lipid membranes (mimicked by liposomes) and the most abundant serum protein, that is, human serum albumin (HSA) [25]. The impact of these molecules on the fluidity of the lipid membrane was monitored by fluorescence spectroscopy (FS) and differential scanning calorimetry (DSC) at increasing dendrimer concentrations. Additionally, the influence of these glycodendrimers on HSA was examined by measuring changes in protein fluorescence intensity (fluorescence quenching, FQ) and alterations in protein secondary structure using circular dichroism (CD) spectrometry. Overall, all generations of **DDMGlus** were found to decrease membrane fluidity and exhibited weak interactions with HSA. Interestingly, unlike other types of dendritic polymers, the extent of interaction between the dendrimers and the biological models was not correlated with dendrimer generation. In particular, **DDM_2_Glu** showed the most significant interaction with the protein, while **DDM_1_Glu** induced the greatest changes in membrane fluidity. Their findings suggest that the flexibility of the hyperbranched molecule, along with its characteristic structure of a hydrophobic interior and hydrophilic surface, as well as the formation of larger aggregates of **DDM_2–3_Glus**, significantly influence the type and magnitude of interaction with biological structures. Given the evidence that the **DDMGlu** dendrimers showed significant interaction with lipid membranes and minimal impact on the model serum protein, the authors concluded that these molecules have a theoretical potential to selectively target cancer cells by exploiting overexpressed glucose transporters.

A collaborative research endeavor involving Spanish and Italian scientists was undertaken to synthesize and characterize a novel series of imidazolium-terminated carbosilane dendrimers and dendrons [26]. The primary focus was to explore their potential as nanocarriers for the delivery of anticancer drugs. The study also aimed to investigate the interaction between these dendrimers and model cell membranes, specifically lecithin-based (LEC) liposomes. To examine the interactions between dendrimers of different generations (G1 to G3, Figure 1b) at various concentrations (1 mM, 10 mM and 20 mM) with cell membrane models, an analysis of the electron paramagnetic resonance (EPR) spectra of selected spin probes was employed. These spin probes were designed to mimic phospholipid behavior and could penetrate the liposome membrane. The EPR analysis provided valuable information on the structure and dynamics of the systems, revealing that higher generations and concentrations of dendrimers led to greater disruption in the liposome structure. The study found that the interaction between dendrons and G1 dendrimers was weak and remained mostly unaffected by dendrimer concentration, gradually diminishing over time. In contrast, G3 dendrimers exhibited significant variations in the interaction process, which depended on their concentration. At low dendrimer concentrations, rapid internalization kinetics were observed. However, the accumulation of dendrimers on the liposome surface hindered this process. The findings of the EPR analysis supported these observations. In addition to EPR analysis, the researchers conducted in vitro experiments to evaluate the cytotoxic effects of the dendrimers and dendrons using prostate adenocarcinoma cells (PC3), triple negative breast cancer cells (HCC1806) and cervical cancer cells (HeLa). The results confirmed the cytotoxic impact of these structures, further highlighting their potential as promising anticancer agents.

A study performed by an Italian and Danish team investigated the interactions between differently functionalized polyamidoamine (PAMAM) dendrimers (bearing amine, acetamide, and 3-methoxy-carbonyl-5-pyrrolidonyl terminal groups) and model membranes (sodium dodecyl sulfate (SDS) and sodium hexadecylsulfate (SHS) micelles, as well as LEC liposomes) [27]. To facilitate analysis, the dendrimers were labeled with the 3-carbamoyl-PROXYL radical. The successful labeling and shielding effects of acetamide and pyrrolidone functions on proton signals from neighboring nitroxide groups were confirmed via ^1^H-NMR (nuclear magnetic resonance) spectroscopy. Through computer-assisted analysis of EPR spectra, it was observed that acetamide-functionalized dendrimers predominantly (60%) interacted with the interface of SDS micelles, while amino-dendrimers exhibited electrostatic interactions with both SDS and SHS surfaces, resulting in dendrimer aggregates in solution. Pyrrolidone-functionalized dendrimers displayed intermediate behavior between amino and acetamide groups (Figure 1c). Weak interactions between acetamide- and pyrrolidone-functionalized dendrimers and LEC liposome surfaces were observed, with a synergistic interplay between hydrophilic and hydrophobic interactions. On the other hand, interactions between liposomes and amino dendrimers were relatively strong, leading to dendrimer aggregation at the liposome surface in the solution. These findings suggest that acetamide- and pyrrolidone-functionalized dendrimers could serve as viable alternatives to amino-dendrimers for drug delivery purposes.

**Figure 2:**
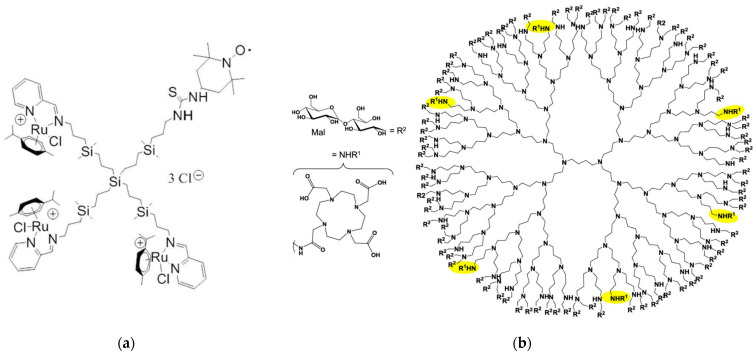
(**a**) Molecular structure of the G1 Schiff-base carbosilane Ru(II) metallodendrimer covalently modified with the EPR spin label TEMPO. Adapted from [28], published by MDPI, 2019. (**b**) Molecular structure of the G4 PPI glycodendrimer, constituted by a dense maltose shell (R^2^) and decorated with six groups of 1,4,7,10-tetraazacyclododecan-N,N′,N″,N‴-tetraacetic acid (DOTA, NHR^1^, highlighted in yellow). Adapted with permission from [30]. Copyright 2020, American Chemical Society.

Schiff-base carbosilane Ru(II) metallodendrimers are potential anticancer agents with a yet undiscovered mechanism of action. Their in situ molecular interactions with model cell membranes (specifically cetyltrimethylammonium bromide (CTAB) micelles and LEC liposomes) were investigated by Carloni et al. using EPR in a Spanish–Italian collaboration [28]. Both a spin probe (4-(N,N-dimethyl-N-dodecyl)ammonium-2,2,6,6-tetramethylpiperidine-1-oxyl (CAT12)) able to enter model membranes, and a spin label (2,2,6,6-tetramethylpiperidine-1-oxyl (TEMPO)) covalently attached to newly synthesized heterofunctional dendrimers, were used to provide complementary information on the dendrimer–membrane interactions (Figure 2a). The computer-assisted EPR analysis revealed an outstanding correlation between spin probe and spin label investigations. Both perspectives highlighted the partial incorporation of dendrimer surface groups into surfactant aggregates, specifically CTAB micelles, as well as the coexistence of polar and hydrophobic contacts, whereas dendrimer–LEC liposome interactions involved more polar contacts between surface groups. The authors also suggested that minor alterations to the structure of dendrimers could drastically alter their ability to interact with cell membranes and consequently their anticancer activity.

Copper (II) ions can have both detrimental and beneficial effects in various diseases, including malignancies, neurological disorders, and Wilson’s disease [29]. Understanding the interaction between Cu(II)-nanodrug and Cu(II)-nanocarrier complexes with cell membranes is therefore a topic of great interest because of their potential application as nanotherapeutics. A study by a German–Italian team explored the complex interaction between 1,4,7,10-tetraazacyclododecan-N,N′,N″,N‴-tetraacetic acid (DOTA)-functionalized poly(propyleneimine) (PPI) glycodendrimers (Figure 2b) and Cu(II) ions, as well as neutral and anionic lipid membrane models using different liposomes [30]. Multiple techniques, including dynamic light scattering (DLS), zeta potential (ZP), EPR, fluorescence anisotropy (FA), and cryogenic transmission electron microscopy (cryo-TEM), were employed to investigate these interactions. The EPR provided insights into the structure and dynamics of the PPI glycodendrimers and their Cu(II) complexes in relation to liposomes. Cu-N_2_O_2_ coordination was found to dominate at the binding site, although it was somewhat weakened at the outer interface due to competitive interactions between the dendrimer and liposomes, resulting in minimal disruption of the liposome structure. Fluorescence anisotropy was utilized to assess the fluidity of the hydrophobic and hydrophilic regions of the lipid bilayer. DLS and ZP measurements were used to determine the size distribution and surface charge of the nanoparticles (PPI glycodendrimer and liposomes), respectively. DOTA-PPI glycodendrimers exhibited selective removal of Cu(II) ions from the bioenvironment, while also preferentially interacting with negatively charged liposomes at the liposome surface. However, these compounds were unable to traverse the model cell membrane and caused minimal disruption to its structure, indicating their potential for various biological applications.

### 2.2. Covalent Dendrimers in Drug Delivery

**Figure 3:**
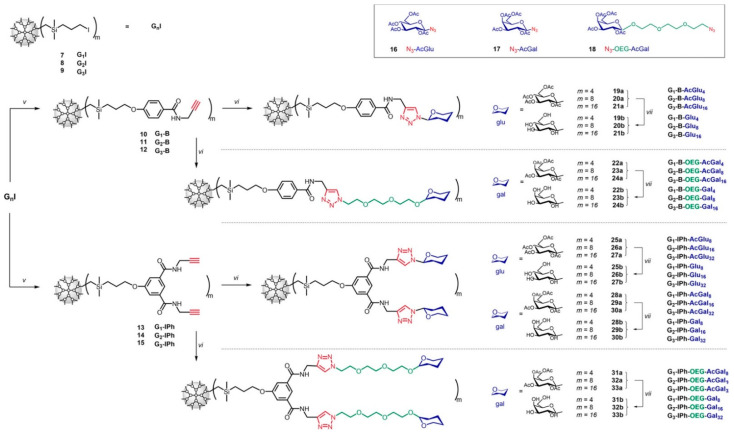
Synthetic pathway leading to the different series of G1–G3 glyco-DDMs. Adapted with permission from [31]. Copyright 2022, American Chemical Society.

A Czech group in collaboration with scientists from Portugal very recently provided new synthetic routes for a series of G1–G3 CSDs that feature glucose, galactose, and oligo (ethylene glycol) modified galactose moieties as surface groups [31]. In this study, the authors presented a robust synthetic method for obtaining stable glycodendrimers (glyco-DDMs) with all the peripheral units mentioned above (Figure 3). The biocompatibility of the glyco-DDMs was evaluated through in vitro cytotoxicity assays, demonstrating excellent results. To explore their potential in drug delivery, the authors encapsulated the anticancer agent doxorubicin (DOX) within the glyco-DDM structure. The anticancer activity of the resulting glyco-DDM/DOX complexes was assessed in both noncancerous (BJ) and cancerous (MCF-7 and A2780) cell lines, revealing a promising effect that was dependent on the generation and concentration of the complexes. In particular, all DDMs exhibited similar entrapment capacities, with 4–6 DOX molecules per DDM independently of generation. The FM analysis indicated that the DOX molecules in the complexes were situated in the interphase between the saccharide-modified outer region and the hydrophobic carbosilane interior. Computational simulations further supported these findings by revealing DOX interactions with both hydrophilic and hydrophobic glyco-DDM domains, indicating their crucial role in the encapsulation process. In vitro cytotoxicity assays of the glyco-DDM/DOX complexes demonstrated generation- and concentration-dependent effects across various cell lines. However, the complexes exhibited preferential selectivity toward cancer cell lines, particularly the A2780 cell line, which was 5–6 times more selective compared to the MCF-7 alternative. Most of the complexes showed higher IC_50_ values compared to pure DOX, suggesting that the peripheral saccharide units significantly reduced the cytotoxicity of the glyco-DDM/DOX complexes. This implies that the glyco-DDM nanocarriers could accommodate higher doses of DOX for improved cancer therapy, while the slower drug release helped maintain a stable concentration of the therapeutic agent within malignant cells. Furthermore, DOX release was found to be two to three times faster in acidic environments compared to physiological conditions (pH = 7.4), indicating preferential drug release in proximity to tumor tissues. Notably, there was a significant difference in cellular uptake, since the glyco-DDM/DOX complexes showed much lower internalization in noncancerous BJ cells compared to both cancerous cell lines. However, more comprehensive research is required to investigate the mechanisms of cellular uptake and trans-barrier trafficking before testing these systems in vivo.

Hydrogels are gel-like structures composed of three-dimensional networks that have the ability to absorb water without dissolving [32]. These materials are characterized by their soft, flexible, and porous nature, and they possess a high-water content, which enhances their biocompatibility. As a result, hydrogels have found wide-ranging applications in the production of contact lenses, hygiene products, and wound dressings, as well as in the fields of drug delivery and tissue engineering [33,34]. Nevertheless, the inherent hydrophilicity of hydrogels limits their compatibility with hydrophobic substances, which constitute approximately 40% of commercially available drugs and 90% of drugs currently being developed [35]. Dendritic macromolecules have recently gained attention as compelling agents for crosslinking in hydrogel synthesis. In addition to the benefits provided by dendrimers, such as monodispersity and multivalency, carbosilane scaffolds are particularly appealing because of their exceptional stability, inertness, and lipophilic properties. These characteristics enhance compatibility with lipophilic cargo and facilitate the nanostructuring of hydrogels, offering additional advantages. However, the nonbiodegradable nature of some dendrimer scaffolds may pose challenges in the pharmaceutical domain. To overcome this limitation, a family of cleavable CSDs was specifically designed for this purpose by a Spanish, American and Italian collaboration [36]. By employing thiolene click-chemistry, biodegradable dendritic hydrogels with low swelling properties and aromatic nanodomains were easily synthesized. Through EPR, molecular dynamics (MD) simulations, and experimental assays, the influence of the CSD nanodomains on the encapsulation and release behavior of model drugs such as ibuprofen (IBU, a well-known nonsteroidal anti-inflammatory drug (NSAID)) and curcumin (CUR, a natural molecule with anticancer activity) was confirmed. Furthermore, the effectiveness of CUR-loaded hydrogels was assessed in vitro against PC3 cells. These dendritic hydrogels not only allowed efficient drug encapsulation, but also demonstrated a proof-of-concept by attaching IBU via fluoride-promoted esterification, enabling controlled release over a specific time interval through enzymatic cleavage.

The therapeutic efficacy of indomethacin (IND), another NSAID with poor water solubility, relies on its efficient transportation across cell membranes and accumulation within target cells. To address this challenge, dendritic polymers have been proposed to enhance the drug’s solubility and intracellular delivery. In their study, a team composed of Polish, German and Danish scientists investigated the anti-inflammatory potential of newly developed PAMAM dendrimers coated with 4-carbomethoxypyrrolidone, which are highly biocompatible and loaded with IND [37]. Their findings demonstrate that the complexation of IND with the investigated dendrimers did not affect the ability of the drug to inhibit the enzymatic action of cyclooxygenases COX-1 and COX-2. Furthermore, these formulations exhibited enhanced anti-inflammatory properties, as evidenced by the increased inhibition of prostaglandin secretion and expression of the nuclear factor kappa-light-chain-enhancer of activated B cells (NF-κB)-associated genes compared to the free drug. Some of these effects were found to be dependent on dendrimer generation, which was expected since higher dendrimer generations have been shown to improve the solubility of poorly soluble drugs. However, lower-generation dendrimers faced limitations in terms of encapsulation efficiency and drug leakage, which hindered their effectiveness as drug carriers. Consequently, for efficient drug delivery purposes, PAMAM dendrimers of the third and fourth generations were typically recommended. The authors also proposed that the observed anti-inflammatory effects of the drug–dendrimer complexes were primarily attributed to enhanced uptake and increased intracellular accumulation of IND. Further data also indicated that a significant portion of pyrrolidone-coated PAMAM macromolecules localized within endolysosomal compartments following endocytosis, while another fraction was transported to the endoplasmic reticulum and Golgi apparatus via recycling endosomes. Therefore, this phenomenon could be ascribed to a faster and more efficient delivery of IND to its intracellular target sites, thus increasing the anti-inflammatory potential of the drug. Although further investigations are required to elucidate the molecular mechanisms underlying these observations in vitro, they provide a foundation for subsequent in vivo studies, potentially leading to the development of novel, biocompatible formulations of IND for clinical applications.

The fourth generation of maltose-modified PPI dendrimers was the subject of a study by a Polish–German team as potential nanocarriers for triphosphate forms of anticancer adenosine analogs in order to improve the effectiveness of chemotherapy and overcome drug resistance mechanisms [38]. This approach has shown success in the administration of fludarabine triphosphate (F-ara-ATP), as the noncovalent complex between the nucleotide and the dendrimer facilitated autonomous cellular entry, leading to increased intracellular accumulation and cytotoxic activity of the drug’s active metabolite. However, when a similar strategy was attempted for clofarabine triphosphate (Cl-F-ara-ATP), the drug’s activity was inhibited. To gain a better understanding of this phenomenon, the authors deemed it necessary to characterize and compare the drug–dendrimer complexes, specifically focusing on the differences in their surface properties and the strength of interactions between the two alternative drugs and the dendrimers. Consequently, ZP measurements, ultrafiltration (UF), and asymmetric flow field-flow fractionation (AF4) techniques were used to determine the surface electrostatic potential and stability of the nucleotide–dendrimer formulations. In aggregate, their experiments revealed that Cl-F-ara-ATP exhibited stronger and more quantitative interactions with the PPI dendrimers compared to those of F-ara-ATP (i.e., more Cl-F-ara-ATP molecules bound to the dendrimer under the same conditions), thereby supporting the hypothesis that the Cl-F-ara-ATP/dendrimer complex displayed reduced drug release, potentially limiting the delivery capability of PPI macromolecules in the case of this drug. According to the authors, variations in the strength of interactions, and consequently the stability of complexes, could be attributed to the structural modifications present in the tested adenosine analogues. In the case of fludarabine and clofarabine, the only differences lie in the substitution of two halide atoms, where fludarabine has a fluorine atom in the 2-position of the adenine base, while clofarabine has a chlorine substitution. Additionally, Cl-F-ara-ATP introduces an extra fluorine atom at the 2′ position of the arabinofuranosyl ring. The higher hydrophilicity of F-ara-ATP may lead to competition between the nucleoside part of the molecule and the phosphate tail for binding to the surface amino groups of PPI dendrimers. Since the phosphate residues of the nucleotide are primarily responsible for interacting with the surface amino groups of PPI dendrimers, this may facilitate the release of this drug from the relevant complex.

Photodynamic therapy (PDT) is an alternative treatment for skin cancer that involves a photosensitizer, light, and oxygen to induce cell death. However, the effectiveness of PDT can be limited by issues such as poor solubility, low tumor selectivity, and inadequate cellular absorption of photosensitizers [39]. Rose Bengal (RB), an anionic dye with potential as a photosensitizer in cancer PDT, faces challenges such as a short half-life, limited transmembrane transport, aggregation, and self-quenching. To overcome these limitations and enable its clinical application, efficient drug carriers are necessary. In a Polish, Portuguese, and Italian collaboration, the researchers conducted comprehensive in vitro and in silico investigations to characterize the interactions between RB and G3–G4 of cationic PAMAM and PPI dendrimers [40]. They evaluated the ability of the resulting complexes to modulate the photosensitizing properties of RB. The binding of RB to the dendrimers was found to be influenced by the generation and structure of the hyperbranched nanocarriers. This interaction enhanced cellular absorption, increased the formation of singlet oxygen and intracellular reactive oxygen species (ROS), and ultimately led to enhanced phototoxicity. Based on their findings, the authors concluded that the use of dendrimer carriers holds promise for the development of effective PDT-based treatments using RB. Interestingly, because of their unique structural properties, interaction patterns with RB, and distinct characteristics of the dendrimer:RB complexes, PPI dendrimers demonstrated superior performance compared to those of PAMAM dendrimers. Specifically, PPI G4 dendrimers exhibited the highest efficiency in uptake, while PPI G3 dendrimers significantly enhanced the generation of singlet oxygen. Accordingly, the authors proposed that these nanovectors can effectively address the challenges associated with RB, paving the way for improved PDT approaches in cancer treatment.

Continuing this trend, the same Polish group in teamwork with French colleagues conducted a preliminary study aimed at evaluating the potential of phosphorus dendrimers as carriers of RB [41]. The synthesis and in vitro investigation of covalent drug–dendrimer conjugates were the main objectives of this research. RB-dendrimer conjugates were generated using a strategy that involved tyramine as an aromatic linker between the carrier and the drug. Various techniques including Fourier-transform infrared spectroscopy (FT-IR), ^1^H NMR, ^13^C NMR, ^31^P NMR, size and ZP studies, and FS analyses were employed to characterize the compounds. The release of the drug from the conjugate was evaluated by dialysis, while intracellular uptake was assessed using flow cytometry (FC). Singlet oxygen production assays were performed, and the biological activity of the compounds was measured using the 3-(4,5-dimethylthiazol-2-yl)-2,5-diphenyl-2H-tetrazolium bromide (MTT) assay. The findings indicate that the tyramine-linked conjugation of RB to phosphorus dendrimers resulted in a reduction in the photodynamic activity of RB. This preliminary work demonstrates the feasibility of using phosphorus dendrimers as carriers for photosensitizers; however, further research is required to optimize the conjugation strategy and enhance the photodynamic activity of RB in the context of PDT.

The synthesis of an aptadendrimer through the covalent bioconjugation of a gallic acid-triethylene glycol (GATG) dendrimer with the G-quadruplex (G4) AT11 aptamer at the surface was carried out by a Spanish, French and Portuguese team [42]. Next, the study focused on investigating the loading and interaction of an acridine orange ligand, C8, which serves both as an anticancer drug and as a binder/stabilizer of the AT11 G4 structure. DLS experiments revealed that the aptadendrimer had a diameter of approximately 3.1 nm. Steady-state and time-resolved FA measurements confirmed the interaction between the aptadendrimer and C8. Interestingly, the iodine atom of the C8 ligand was observed to act as an effective intramolecular quencher in the solution, but when complexed with the aptadendrimer, it adopted a more extended conformation. Molecular modeling studies provided additional support for this finding. The release experiments demonstrated the delivery of C8 from the aptadendrimer after 4 h. CFM revealed the localization of aptadendrimers in the cytoplasm of various healthy and malignant prostate cell lines (i.e., PNT1A, PC3, and DU145). The internalization of the aptadendrimers occurred through endocytosis rather than by nucleolin-mediated uptake or passive diffusion. MTT studies conducted on prostate cancer cells and non-malignant cells demonstrated significant cytotoxicity, primarily attributed to the C8 ligand. Accordingly, the rapid internalization of aptadendrimers and their fluorescence properties make them promising candidates for the further development as potential nanocarriers.

### 2.3. Covalent Dendrimers as Anticancer Drugs Per Se

**Figure 4:**
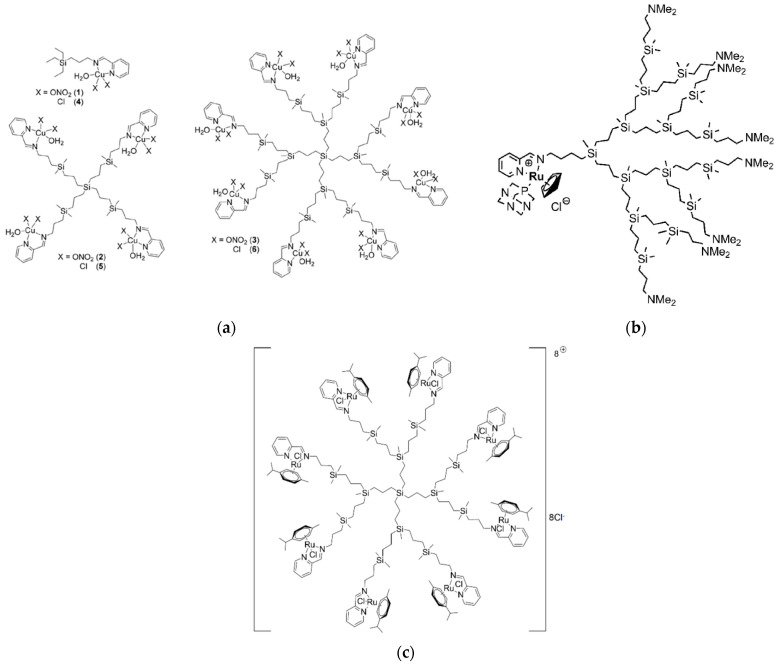
(**a**) Molecular structure of G1 and G2 Cu(II) carbosilane metallodendrimers with nitrate (**1**–**3**) or chloride (**4**–**6**) bound ligands. Adapted with permission from [43]. Copyright 2019, The Royal Society of Chemistry. (**b**) Molecular structure of the G3 heterofunctional Schiff base carbosilane metallodendron featuring the [Ru(5-C5H5)(PTA)Cl] (PTA = 1,3,5-triaza-7-phosphatricyclo-[3.3.1.1]decane) central component. Adapted with permission from [44]. Copyright 2020, Elsevier. (**c**) Molecular structure of the G2 cationic carbosilane ruthenium (II) dendrimers (**CRD27**). Adapted from [46], published by MDPI, 2020.

Sanz del Olmo and other colleagues from Spain and Italy created a brand new family of Cu(II) carbosilane metallodendrimers that are water-soluble as a nanotechnological replacement for conventional therapies [43]. Interactions between dendrimers of various generations (G0 to G2) with two Cu(II) counterions (nitrate vs. chloride) (Figure 4a), and with two cell membrane models (namely, CTAB micelles and LEC liposomes), were examined over time using computer-aided analysis of EPR spectra. The systems’ structural and dynamical information from EPR analysis showed that the relative amount and intensity of interaction between the dendrimer and the model membranes increased by the rise in generation and the switch from nitrate to chloride for the Cu(II) counterion. Interestingly, the stabilizing impact decreased the toxicity of cancer cells. Using a variety of different cell lines, including a human fibroblast cell line (142BR) representing healthy cells, as well as tumor cell lines derived from the cervix (HeLa), both normal and drug-resistant breast cancer cells (MCF-7 and HCC1806), advanced prostate cancer (PC3), and colorectal tumor (HT29), the selective lethal effect of Cu(II) metallodendrimers on malignant cells was demonstrated in vitro, confirming the impact of multivalency on the potency and selectivity of the metallodrugs. For instance, the tetrametallic dendrimer G1-Cu(ONO_2_) exhibited significantly higher cytotoxicity against the HeLa cell line compared to the monometallic counterpart, with a 28-fold increase. Notably, the Cu(II)-metallodendrimers effectively eradicated drug-resistant tumor cells, displaying IC_50_ values below 3.4 μM in PC3 cells and below 1.9 μM in the HCC1806 cell line. The first-generation dendrimer G1-Cu(ONO_2_)_2_ was finally selected for a detailed evaluation of in vitro and in vivo anticancer activity against prostate malignancies with acquired resistance as a proof-of-concept. The Cu(II)-metallodendrimers considerably decreased tumor growth during the experiments without displaying any toxicity, indicating their promising potential as anticancer metallodrugs.

In a subsequent effort, the same Spanish group in collaboration with Polish colleagues developed a new series of heterofunctional Schiff base carbosilane metallodendrons consisting of a central component [Ru(5-C_5_H_5_)(PTA)Cl] (PTA = 1,3,5-triaza-7-phosphatricyclo-[3.3.1.1]decane) and bearing dimethylamino groups attached to their outer surface (Figure 4b) [44]. The researchers found that these novel compounds were capable of interacting with biological molecules such as HSA without causing any changes to its secondary structure. Additionally, when exposed to erythrocyte membranes, the metallodendrons caused a degree of hemolysis proportional to the dosage and generation of the compound. The presence of two active functional groups on a single dendritic platform had a synergistic effect on the effectiveness of the metallodendrons against HeLa and PC3 cancer cell lines. The G2 derivative of the compound exhibited the most potent anticancer activity, with the lowest IC_50_ value (1.4 μM) indicating a higher effectiveness. Further experiments focused on advanced prostate cancer revealed that the metallodendrons could inhibit the migration and adhesion of cancer cells to the bone, which is one of the primary sites of metastasis for this type of cancer. To investigate the effects of metallodendrons on advanced prostate cancer in a controlled environment, the researchers selected the second generation metallodendron with a single metal core and four dimethylamino groups on the dendritic surface for an ex vivo study. The study involved using nude mice with advanced malignancy. The results showed that compared to the control animals, the selected metallodendron inhibited tumor growth by 40%.

A multinational study involving Spanish, American, Slovakian and Polish researchers focused on the development of Ru-based metallodendrimers labeled with a fluorescent marker (**FITC-CRD13**) and their coupling with graphene oxide-modified gold nanowires (GOAuNW) [45]. The objective was to evaluate these complexes as active intracellular transporters, specifically using the MCF-7 breast cancer cell line as a model. The study also explored the influence of an ultrasonic field (USF) in driving the movement of these complexes. To confirm the successful modification of GO-AuNWs by dendrimers, energy-dispersive X-ray spectroscopy (XEDS) analysis was conducted. The analysis demonstrated that ruthenium was uniformly distributed over the nanomotor structure, indicating the presence of **FITC-CRD13**. The binding of dendrimers to the surface of GO-AuNWs resulted in a suppression of the fluorescence signal. However, when an USF was applied for 5 min at 2 volts and 2.66 MHz, the complexes were propelled towards the cancer cells. As a result, they separated from the GO-nanomotor surface and regained their dendrimer fluorescence signal. The fluorescence signal of the samples treated with US was 1.8 times higher than that of the passive controls. Based on these findings, the authors concluded that the use of ultrasound-propelled AuNWs leads to enhanced cell internalization, thus increasing the delivery of carbosilane ruthenium dendrimers to MCF-7 cells.

In a follow-up work, the same Spanish group in collaboration with coworkers from Poland and Belarus investigated the antitumor activity of the first and second generation of carbosilane dendrimers (**CRD13** and **CRD27**, respectively), this time functionalized with Ru(II) complexes at the periphery (Figure 4c) against the human acute lymphoblastic leukemia (ALL) 1301 cell line as a proof-of-concept using various techniques such as TEM, the comet test, and real time polymerase chain reaction (rtPCR) [46]. The levels of ROS and changes in mitochondrial potential were also assessed. The results demonstrated that these ruthenium dendrimers significantly reduced the viability of the leukemia cells while exhibiting minimal toxicity towards noncancerous cells (peripheral blood mononuclear cells (PBMCs)). Examination of 1301 cells using TEM demonstrated that treatment with **CRD13** and **CRD27** dendrimers induced alterations in cell morphology and structure. The cytoplasm exhibited increased density and the cell surface displayed a higher number of vesicular structures. In particular, the micrographs captured in this study revealed alterations in the cell ultrastructure of 1301 cells upon treatment with **CRD13**. These changes include the presence of multiple multivesicular bodies, lamellar bodies, lipid deposits, and swollen mitochondria with small vacuoles, all features typically associated with early apoptosis.

Back in the field of Cu(II) carbosilane metallodendrimers, it was hypothesized that specific structural features, such as dendrimer generation and metal counterion, would influence the interaction with tumor cells, thereby affecting treatment efficacy and selectivity. By employing computer-assisted analysis of EPR spectra, the results of a joint Spanish–Italian effort yielded valuable dynamic and structural information on the interactions between the G0–G3 of these dendrimers (Figure 4a) and tumor cells (the pro-monocytic, human myeloid leukemia cell line U937), as well as PBMCs over time [47]. To investigate the fate of metallodendrimers within cells, a comprehensive in vitro assessment was conducted that included cytotoxicity, cytostaticity, and sublethal effects on mitochondrial function, lysosomal compartments, and participation in autophagic organelles. The EPR results indicated that chloride dendrimers and low-generation complexes exhibited greater membrane stability, which ultimately influenced the uptake and intracellular fate of the metallodrugs. Cu(II) metallodendrimers demonstrated cytostatic effects and moderate cytotoxicity against U937 tumor cells. These molecules induced apoptosis through the mitochondria-lysosome pathway and led to the formation of autophagic vacuoles. Notably, these metallodrugs had minimal impact on healthy monocytes in vitro. Overall, these results provide valuable insights into the mechanism of action and highlight the crucial structural characteristics that influence the activity of these nanoscale metallodrugs.

In a successive effort, again starting from the dendrimer family shown in Figure 4a, the same team successfully synthesized and comprehensively characterized new Cu(II) carbosilane metallodendrimers containing methyl or methoxy substituents on the pyridine ring, with a (simplified) general formula **Gn-RL**, where n indicates the dendrimer generation (n = 0, 1, 2), R the ring substituent (R = Me, OMe) and L is the copper counterion (L = Cl, NO_3_) [48]. Through the use of EPR, the researchers revealed the distinct coordination modes of the Cu(II) ion (Cu-N_2_O_3_, Cu-N_4_, and Cu-O_4_), whose proportions were influenced by the structural features of the dendritic molecules. These metallodendrimers exhibited IC_50_ values in the low micromolar range (6 μM) against HeLa and MCF-7 cancer cell lines. Further in vitro investigations on both healthy (PBMC) and malignant (U937) myeloid cells identified two crucial factors that enhanced the cytotoxicity and selectivity of the metallodrug. Firstly, optimizing the Cu-N_2_O_2_ coordination mode and secondly, selecting the appropriate ring-substituent/metal counterion pair. The most promising candidates, i.e., **G1(-CH_3_)Cl** and **G1(-OCH_3_)NO_3_**, demonstrated a significant increase in anticancer activity against U937 tumor cells compared to their non-substituted counterparts, potentially through two distinct routes involving ROS production.

In an Italian–Bulgarian cooperative work, the activity and interaction ability of a PAMAM dendrimer modified with 4-N-methylpiperazine-1,8-naphthalimide units (referred to as D) and complexed with Cu(II) ions against both healthy and malignant cancer cells, was investigated [49]. In this study, the authors conducted comparative EPR analyses of the Cu(II)-D complex, examining its coordination mechanism, chemical structure, flexibility, and stability in the presence and absence of myeloid U937 cancer cells and PBMCs. The interactions of Cu(II) ions in biological media at different equilibrium periods provided insights into their stability and contact with cells. FC and CMF were employed to characterize the unique properties of the dendrimers in PBMC and U937 cells. Additionally, they used a novel fluorescent probe (referred to as Fly) for potential biological imaging of Cu(II). This research demonstrates that the dendrimer and Cu(II) metallodendrimer exhibit cytotoxic effects on cells, particularly U937 tumor cells, leading to mitochondrial dysfunction, increased generation of ROS, and involvement of lysosomes. The anticancer selectivity of the metallodendrimer, which minimally affects healthy PBMC while inducing significant apoptotic cell death in U937 cells, aligned with the high stability of this complex as observed in EPR investigations, highlights the potential of this metallodendrimer as a promising candidate for anticancer therapy.

### 2.4. Covalent Dendrimers in the Delivery of Nucleic Acids

Gene therapy is an increasingly promising approach to therapeutics, as it offers the ability to specifically target disease-causing genes, allowing the precise and personalized treatment of various life-threatening conditions. By introducing specific nucleic acids (NAs) into the targeted tissues of patients, gene expression can be modulated by down-regulating, augmenting, or correcting gene activity. Different molecules such as small interfering RNA (siRNA), microRNA (miRNA), messenger RNA (mRNA), small activating RNA (saRNA), inhibitory antisense oligonucleotides (ASOs), splicing-modulatory ASOs, plasmid DNA, and CRISPR/Cas systems are commonly utilized to achieve these goals. These modalities have shown promise in numerous therapeutic programs aimed at treating specific diseases [50,51]. Despite the promising potential of NAs in drug development, its widespread clinical application is hindered by various intracellular and extracellular obstacles. The use of naked and unmodified NAs presents certain drawbacks, including (1) inadequate stability and unfavorable pharmacokinetic properties, and (2) the potential for inducing off-target effects among several others [52]. To address the challenges associated with safe and efficient NA delivery, researchers have devised viral-vector-based and nonviral delivery systems that aim to safeguard NAs from degradation, enhance targeted delivery to specific cells, and minimize exposure to off-target cells. While viral gene therapies have demonstrated promising clinical outcomes, their effectiveness is often constrained by factors such as preexisting immunity, viral-induced immunogenicity, unwanted genomic integration, limitations in payload size, the inability to administer repeat doses, complexities in scaling up production, and the high cost of vector manufacturing [53]. Although efforts are being made to overcome some of these limitations, scientists have prompted the exploration of alternative drug delivery vehicles. In parallel, advances in the development of synthetic materials, including polymers, lipids, lipid nanoparticles (LNPs), and dendrimers, have revived research into nonviral delivery systems for the encapsulation of NAs [2].

**Figure 5:**
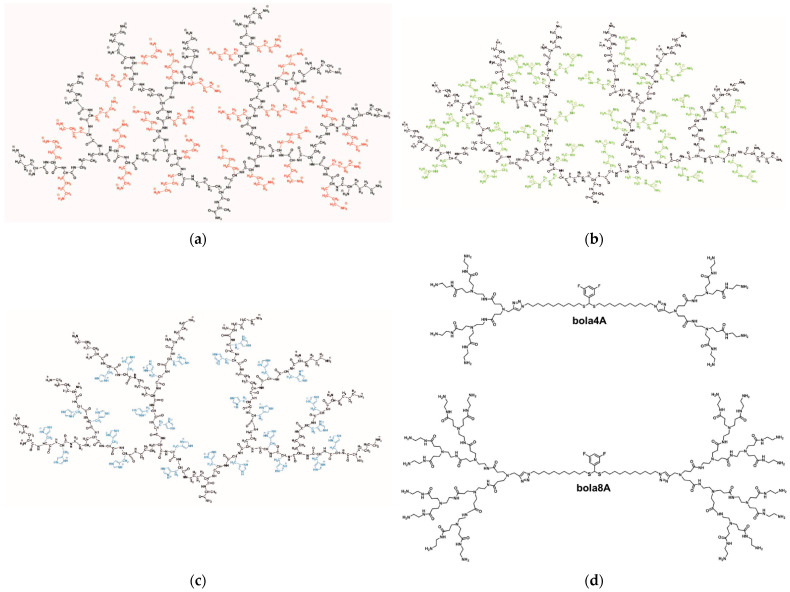
Molecular structures of poly(lysine) dendrimers with lysine (**D3K2**) (**a**), arginine (**D3R2**) (**b**), and histidine (**D3H2**) (**c**) as side groups of spacers between each pair of neighboring branching points. Adapted from [54], published by MDPI, 2020. (**d**) Molecular structures of the two amphiphilic dendrimers **bola4A** and **bola8A**. Adapted with permission from [56]. Copyright 2023, The United States National Academy of Sciences.

In this fascinating but challenging scenario, a Polish, Russian, and German team performed a study aimed to investigate the formation of siRNA dendriplexes using novel poly(lysine) dendrimers (PLDs) containing lysine (**D3K2**) and either arginine (**D3R2**) or histidine (**DRH2**) residues (Figure 5a–c) [54]. The primary objective of this study was to conduct an initial characterization of the dendrimer–siRNA complexes and assess their in vitro transfection efficiency, with the final objective of identifying optimal conditions for subsequent research. The dendrimer–siRNA complexes were analyzed using fluorescence polarization (FP) experiments, ZP evaluations, and hydrodynamic diameter measurements. The cytotoxicity of dendrimers and dendriplexes was assessed using a resazurin-based assay. FC was used to evaluate the efficacy of siRNA transportation to human THP-1 (acute monocytic leukemia) and U937 cell lines. Through these analyses, the characteristics and optimal molar ratios of the dendrimer–siRNA complexes were determined. The study demonstrated that poly(lysine) dendrimers, compared to the commercial transfection agent Lipofectamine 2000, exhibited excellent carrier capabilities for genetic material. They were more effective in transporting siRNA to cells and showed lower cytotoxicity. These findings lay the groundwork for future investigations into the use of PLDs as NAs carriers in gene therapy applications.

The same group continued their research by further synthesizing two types of hyperbranched molecules, i.e., **D3K2**, which contained additional lysine residues (Lys-Lys) with charged NH_3_^+^ groups at specific branching points, and **D3G2**, which contained additional glycine residues (Gly-Gly) at the same points [55]. These modifications were introduced to enhance interactions with genetic material by increasing the positive charge (**D3K2**) or flexibility (**D3G2**) of the dendrimer. The cytotoxicity of **D3K2** and **D3G2** was evaluated, along with their gene delivery potential, to identify the concentration range that maintains a proper balance between cytotoxic activity and transfection efficiency for further investigations. The study focused on the two HeLa and HMEC-1 cell lines (microvascular endothelial cells), representing cancerous and normal cells, respectively. Cytotoxicity assessments using MTT and crystal violet assays revealed that both **D3K2** and **D3G2** dendrimers exhibited minimal toxicity against HMEC-1 cells. However, HeLa cells displayed higher sensitivity to these macromolecules, with **D3K2** demonstrating greater time- and concentration-dependent toxicity compared to **D3G2**. This observation is consistent with cationic dendrimers’ tendency to interact nonspecifically with cellular membranes, induce mitochondrial damage, and stimulate reactive oxygen species ROS production, leading to enhanced cytotoxic activity. The selective cytotoxicity of PLDs toward the HeLa cell line is particularly significant, as it suggests their potential application as anticancer drug carriers or standalone therapeutic agents, which could offer synergistic effects and reduce the adverse effects associated with conventional chemotherapy. Furthermore, a DNA comet assay was used to evaluate the impact of the investigated compounds on NA degradation. The results aligned with the cytotoxicity evaluation, showing significantly higher levels of DNA damage in HeLa cells treated with **D3K2** dendrimer compared to HMEC-1 cells, especially after longer incubation periods. These findings suggest the involvement of apoptotic processes in the death of cells treated with PLDs or direct genotoxic activity, as the comet assay cannot distinguish between these two phenomena. To determine whether DNA degradation observed in the comet assay was associated with cell death induced by apoptosis, a TUNEL assay was conducted on HeLa cells, which exhibited higher levels of DNA damage induced by **D3K2** compared to **D3G2**. The TUNEL assay demonstrated a higher percentage of cells with DNA damage than the comet assay, as the latter cannot efficiently detect cells in later stages of apoptosis. Notably, the time-dependent increase in DNA damage observed in the comet assay was not observed in the TUNEL assay, suggesting that poly(lysine) dendrimers may primarily induce apoptosis (e.g., through increased ROS production) rather than exert genotoxic effects. Transfection efficiency was assessed using pcDNA3-EGFP, a large plasmid, in both cell types using **D3K2** dendrimer as an example. **D3K2** dendrimer was able to introduce the plasmid into cells with comparable or even superior efficiency to commercially available lipofectamine, particularly in HeLa cells. However, in HMEC-1 cells, the limited toxicity of **D3K2** did not affect transfection efficacy, suggesting no correlation between these two phenomena. Additionally, the dendriplexes exhibited lower toxicity than lipofectamine in both cell lines, especially after longer incubation periods, indicating the high transfection potential of **D3K2** dendrimer across various cell types. Conversely, **D3G2** dendrimer showed minimal transfection capacity, highlighting the greater importance of positive charge rather than flexibility in this context. Furthermore, the higher transfection efficiency achieved with increasing **D3K2** concentration suggests a concentration-dependent cellular uptake of cationic dendrimers, possibly due to variations in cell membrane permeability.

In a very recent study involving French, Italian, and Chinese scientists, two bola-amphiphilic dendrimers—**bola4A** and **bola8A** (Figure 5d)—were designed and synthesized as a customized platform for selective and controlled delivery of DNA and siRNA, respectively, in precision cancer treatment [56]. These dendrimers surpassed current gold standard vectors and indeed demonstrated cargo-selective delivery. Specifically, the G3 dendrimer **bola8A** exhibited superior DNA delivery performance compared to the G2 dendrimer **bola4A**. On the other hand, **bola4A** was more efficient at delivering siRNA than **bola8A**. The discrepancy in nucleic acid delivery performance between the two hyperbranched molecules was attributed to differences in the sizes of the nucleic acid cargoes and the generation number of the dendrimers. Indeed, due to its longer sequence and larger size, DNA requires the higher-generation dendrimer **bola8A** for effective delivery. The increased number and strength of cooperative and multivalent interactions provided by **bola8A** facilitate the compaction of large DNA cargos into smaller nanoparticles, promoting their cellular uptake and successful delivery for gene transfection. Consequently, **bola8A** outperformed **bola4A** in DNA delivery. In contrast, smaller siRNA cargo can be easily encapsulated by **bola4A** and **bola8A**, resulting in siRNA delivery complexes of similar size and surface potential. This similarity enables comparable cellular uptake and escape from endosomes. However, the release of siRNA from the siRNA/**bola4A** complex was more efficient due to the smaller dendrons in **bola4A**, which offer a more balanced and effective cooperative multivalent interaction for the binding and release of siRNA. Therefore, **bola4A** was more effective than **bola8A** in siRNA delivery. Remarkably, both bola dendrimers combine the advantages of lipid and polymer vectors and incorporate tumor-targeting capabilities and ROS-responsive cargo release in cancer cells. This combination led to excellent performance in the delivery of siRNA and DNA specifically to tumor cells in various cancer models, including aggressive and metastatic malignancies, both in vitro and in vivo. When delivering RAC-beta serine/threonine protein kinase (AKT2) siRNA and p53 plasmid DNA using **bola4A** and **bola8A**, respectively, effective anticancer activity and therapeutic effects were achieved in mouse models of ovarian and cervical cancer xenografts (SKOV-3 and HeLa), as well as lung metastasis models induced by triple negative breast cancer (4T1) and melanoma (B16-F10) in mice. These results provide experimental evidence supporting the use of custom dendrimer vectors for tissue- and cell-specific delivery of nucleic acid therapeutics in precision cancer treatment. The authors finally observed that the generation number of bola-amphiphilic dendrimers not only influenced cargo selectivity for nucleic acid delivery, but also impacted their toxicity profiles. Both **bola4A** and bola8A exhibited negligible toxicity. These findings should encourage further exploration and development of dendrimer vectors for nucleic acid delivery.

The presence of tumor cells with stem cell properties has been implicated in the progression and aggressive behavior of cancer, necessitating the development of effective therapeutic strategies to target these cells [57,58]. In this perspective, the suitability of polycationic phosphorus dendrimers as delivery vehicles for siRNAs in in vitro models of stem-like cells was the subject of a study performed by a French, German, Russian, and Chinese team [59]. Specifically, Knauer and colleagues focused on Lyn, a member of the Src family of tyrosine protein kinase, as a potential therapeutic target due to its association with negative clinical outcomes in glioma. The authors successfully delivered anti-Lyn siRNA using these dendrimers into Lyn-expressing glioma cell models, resulting in reduced cell viability. In particular, this effect was not observed in cell models lacking Lyn expression. Furthermore, they observed that the dendrimer itself influenced various cell parameters, including the expression of surface markers such as programmed death-ligand 1 (PD-L1), T-cell immunoglobulin and mucin domain-containing protein 3 (TIM-3), and cluster of differentiation 47 (CD47), which are recognized by the immune system, as well as other biological processes related to the invasion of glioblastoma cells. These findings underscore the potential of dendrimer-based platforms for therapeutic applications, particularly in the targeting of cancer cells that exhibit enhanced resistance to chemotherapy.

A collaborative Spanish and Polish research aimed to investigate the interaction between the Schiff-base carbosilane Cu(II) metallodendrimers shown in Figure 4a and pro-apoptotic siRNAs [60]. The nanocomplexes formed by combining the metallodendrimers with various siRNAs were extensively characterized using techniques such as ZP measurement, size analysis, TEM, FS, CD, and electrophoresis. The internalization of dendriplexes in MCF-7 human breast cancer cells was assessed using FC and CFM, providing information on the potential of these metallodendrimers as siRNA carriers. In vitro studies that examined cell survival demonstrated effective interactions between Cu(II) dendrimers and pro-apoptotic siRNAs targeting the induced myeloid leukemia cell differentiation protein (Mcl-1) and the B cell lymphoma 2 proteins (Bcl-2)—two apoptotic regulators—in breast cancer cells. The combination of first-generation derivatives with chloride counterions and siRNA further improved the anticancer efficacy of the dendriplex constructs, establishing them as efficient carriers for pro-apoptotic siRNAs, thus enhancing their therapeutic prospects for cancer treatment.

## 3. Self-Assembling Dendrimers

### 3.1. Self-Assembling Dendrimers in Drug Delivery

Self-assembly refers to the process by which individual units of a material come together and form highly organized structures or patterns. This phenomenon occurs through noncovalent interactions and imparts unique properties to both inorganic and organic structures. Self-assembled nanomaterials are currently being utilized in various fields such as nanotechnology, imaging techniques, biosensors, and biomedical sciences. This is due to the advantages they offer, including simplicity, spontaneity, scalability, versatility, and cost effectiveness. The self-assembly of amphiphiles leading to the formation of nanostructures such as micelles, vesicles, and hydrogels is driven by various physical interactions. Recent advances in drug delivery have provided new opportunities for the development of innovative drug delivery systems, and self-assembled nanostructures have demonstrated great potential as convenient and effective materials for this purpose [61,62,63].

**Figure 6:**
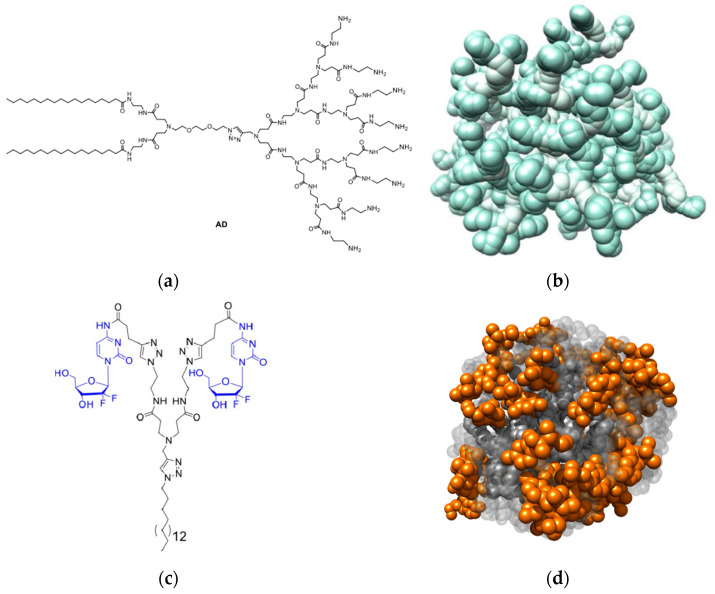
(**a**) Chemical structure of the amphiphilic dendrimer **AD** (right) and (**b**) the computer-derived molecular model of a nanomicelle formed by **AD** upon self-assembly. Adapted with permission from [64]. Copyright 2023, Elsevier. (**c**) Chemical structure of the gemcitabine (Gem) conjugated amphiphilic dendrimer and (**d**) a zoomed snapshot of a nanomicelle formed upon its self-assembly (**GemNM**) as extracted from the corresponding equilibrated MD simulation. Adapted with permission from [65]. Copyright 2022, Elsevier. In (**b**), the charged terminal groups are highlighted in a darker green shade while in (**d**) the Gem moieties are portrayed in orange (in both panels, hydrogen atoms, water molecules, ions and counterions have been omitted for clarity).

In this multifaceted and necessarily multidisciplinary field, an Italian–French team successfully encapsulated two clinically approved anticancer drugs, dabrafenib (DAB) and vemurafenib (VEM), which target the mutated oncogene BRAF in patients with melanoma, within nanomicelles [64]. These nanomicelles were formed through the self-assembly of an amphiphilic dendrimer called **AD**, comprising two C_18_ aliphatic chains and a G2 PAMAM head (Figure 6a). The encapsulation process resulted in the formation of well-defined core–shell nanomicelles with a size of approximately 10 nm (Figure 6b). The nanomicelles exhibited excellent encapsulation efficiency, with around 70% for DAB and 60% for VEM, and a good drug loading capacity of approximately 27% for DAB and 24% for VEM. To characterize the nanomicelles and understand their interactions with the drugs, various techniques were employed, including DLS, TEM, small-angle X-ray scattering (SAXS), NMR, isothermal titration calorimetry (ITC), and MD simulations. These investigations provided insight into the size, structure, and interactions between the nanomicelles and the drug molecules. In vitro release studies indicated that the drug release profiles followed Fickian diffusion, with a more pronounced release under acidic conditions (pH = 5.0) compared to the physiological pH (pH = 7.4) for both DAB and VEM. Additionally, the DAB- and VEM-loaded nanomicelles exhibited enhanced therapeutic efficacy compared to free drug treatments when tested in four different melanoma cell lines. Overall, the successful encapsulation of DAB and VEM within the nanomicelles, along with their controlled release and improved therapeutic response in melanoma cell lines, demonstrates the potential of these nanomicelles as promising delivery systems for targeted anticancer therapy.

Within the framework of a French, Italian, and Chinese partnership, Cong et al. developed an amphiphilic dendrimer-based self-assembling prodrug based on the anticancer drug gemcitabine (Gem) (Figure 6c) [65]. This prodrug exhibits a remarkable drug loading capacity (40%) and has the ability to self-assemble into small, spherical nanomicelles (**GemNMs**) characterized by an average diameter of 7 nm (as determined by DLS, TEM and MD simulations), a negative ZP value of −20 mV (which facilitates electrostatic repulsion and prevents NM aggregation), the absence of backfolding, and with all Gem moieties situated on the micellar outer surface (Figure 6d). These nanomicelles enhanced the metabolic stability of Gem by withstanding the rapid enzymatic conversion of the anticancer drug to its inactive form (dFdU) by cytidine deaminase (CDA), promoted cellular uptake through endocytosis, and bypassed transport-mediated absorption. Moreover, the prodrug nanosystem demonstrates the pH- and enzyme-responsive release of Gem, resulting in improved anticancer efficacy and reduced toxicity in several malignant cell lines, including MCF-7 and three human pancreatic cancer cell lines (PANC-1, Mia-PaCa-2 and SW1990). Ultimately, the anticancer effectiveness of **GemNMs** was tested in vivo by conducting experiments in SW1990 xenograft mice. The results demonstrate that **GemNMs** significantly suppressed tumor growth along with good tolerability compared to the control groups treated with saline or Gem alone, highlighting the superior anticancer activity of **GemNMs** compared to the original Gem drug and thus opening new possibilities for the development of self-assembling prodrugs using amphiphilic dendrimer nanoplatforms.

Pancreatic ductal adenocarcinoma (PDAC) is a devastating malignancy with limited therapeutic options and for which nanomedicine may offer a new ray of hope for treatment. In this regard, the same European Asian team developed a new supramolecular dendrimeric nanosystem containing the anticancer drug DOX, which demonstrated significant anticancer activity and overcame the challenges posed by the heterogeneous response to treatment and resistance of primary cultivated tumor cells derived from tumors of patients with PDAC [66]. The amphiphilic dendrimer nanodrug (**FAD**) was composed of a fluorinated C_18_ alkyl chain as the hydrophobic part and a hydrophilic head of G2 PAMAM dendron, and was able to self-organize into nanomicelles, effectively encapsulating DOX with high loading and encapsulation efficiency (36% and >90%, respectively). This **FAD**-DOX nanosystem exhibited efficient tumor accumulation because of its small (9 nm) nanoparticle size, stable formulation, long circulation time, and acid-triggered drug release. Significantly, this dendrimer nanodrug demonstrates consistent and potent responses in 35 patient-derived primary pancreatic cancer cell lines (of which 15 cell lines were from patients who survived less than 8 months, and the remaining 20 cell lines were from patients who survived more than 8 months), with IC_50_ values well below 10 μg/mL. This remarkable performance surpassed that of the clinical drugs DOX and Caelyx, a clinically available nanoformulation of DOX used as a reference control. This underscores the ability of **FAD**-DOX to effectively overcome the heterogeneity of drug response and drug resistance commonly observed in primary cultured tumor cells derived from PDAC patients. Furthermore, in patient-derived pancreatic tumor xenografts, **FAD**-Dox achieved complete suppression of tumor growth without any notable adverse effects.

**Figure 7:**
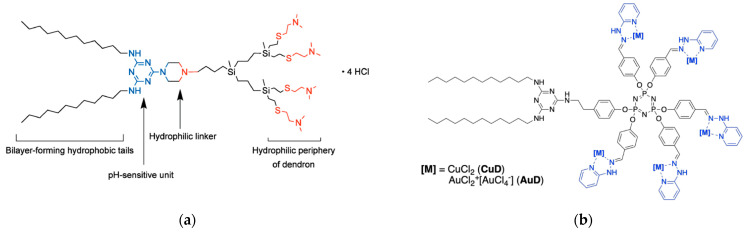
(**a**) Chemical structure of the triazine-carbosilane amphiphilic dendron that generate dendrimersomes upon self-assembly. Adapted from [67], published by MDPI, 2020. (**b**) Chemical structure of the amphiphilic triazine-phosphorous metallodedron featuring multiple Cu(II) or Au(III) complexes as terminal units. Adapted from [68], published by MDPI, 2022.

A novel class of amphiphilic dendritic structures, called triazine-carbosilane dendrons (Figure 7a), has been created by a group of Spanish, Russian, and French colleagues, capable of self-assembling into pH-sensitive vesicle-like nanostructures called dendrimersomes (TCSDSs) [67]. Upon their formation at a neutral pH, the TCSDSs exhibit an average diameter ranging from 20 to 30 nm. However, when exposed to an acidic environment (pH < 6.5), they undergo reorganization and form larger particles with a diameter of 100 to 150 nm while preserving their vesicle-like structure, as confirmed by TEM imaging. These TCSDSs have demonstrated the ability to encapsulate anticancer drugs such as DOX and methotrexate (MTX) with a high drug encapsulation efficiency (~65% for DOX and ~75% for MTX, respectively) and comparable drug loading (~20%). In vitro, the authors observed that TCSDSs loaded with chemotherapeutic agents exhibit a cytotoxic effect on the leukemia cell lines 1301 and K562. Importantly, the cytotoxic effect is dependent on the dose of the encapsulated drugs. Furthermore, the presence of serum has not been found to hinder the ability of these dendrimersomes to penetrate cells or their cytostatic activity, highlighting their potential effectiveness in the presence of biological fluids.

Continuing their research, an international team, now including contributions from German scientists, synthesized amphiphilic triazine-phosphorus amphiphilic dendrons with multiple Cu(II) or Au(III) complexes on the periphery (Figure 7b) [68]. In an aqueous solution, these metallodendrons have the ability to self-assemble into either individual micelles with an average diameter of 9 nm or multi-micellar aggregates with an average diameter of 60 nm. The team investigated the efficacy of these metallodendrons as potential anticancer agents, focusing on glioblastoma, a highly resistant malignant brain tumor. The cytotoxic activity of the metallodendrons was evaluated against glioblastoma stem cells (BTSC233, JHH520, NCH644 and SF188 cell lines) as well as U87 glioblastoma cells. The results revealed that the metallodendrons showed greater cytotoxic activity against glioblastoma cells compared to temozolomide, the current standard of care for this malignancy. Specifically, the copper-containing dendron showed an IC_50_ range of 3–6 μM, while the gold-bound dendrons showed an IC_50_ range of 11–15 μM. On the contrary, the IC_50_ value for temozolomide exceeded 100 μM. These findings emphasize the potential of nanoformulations based on metallodendrons as effective anticancer agents, particularly in the context of the treatment of glioblastoma.

The same triazine-carbosilane dendrons depicted in Figure 7a were used by a collaborative research team consisting of scientists from Poland, Germany, France, Spain, and Russia [69]. The objective of their study was to utilize these dendrons to encapsulate the photosensitizer RB within the corresponding dendrimersomes for potential PDT applications. The team conducted a comprehensive characterization of this novel nanosystem, employing various analytical and spectroscopy techniques to assess its properties. Using established protocols, the researchers successfully synthesized dendrons with precisely tailored characteristics. Biophysical techniques, including measurements of the hydrodynamic diameter, ZP, spectral properties, and TEM, confirmed the formation of drug-encapsulated nanovesicles. To evaluate the functionality of the TCSDSs, a range of in vitro techniques were employed, such as assessing ROS generation, evaluating cell viability using the MTT assay, and examining cellular uptake using FC and CFM. The encapsulation of RB within TCSDSs significantly enhanced cellular uptake, intracellular ROS production, and subsequently increased the phototoxicity of the photosensitizer. These findings highlight the potential of these nanosystems as high-capacity drug carriers for PDT in anticancer treatments.

### 3.2. Self-Assembling Dendrimers in the Delivery of Nucleic Acids

Microglia, which are macrophages residing in the brain, have been implicated in various brain disorders [70]. In light of this, a collaborative team from France, Italy, and Poland conducted a study to evaluate the capability of the amphiphilic dendrimer **AD** (Figure 6a) in transporting siRNA and inhibiting gene expression in primary microglia [71]. The researchers examined the ability of **AD** to form siRNA nanoparticles, assessed their size, surface charge, cellular uptake, and their effectiveness in silencing genes in rodent microglia. In detail, to investigate the response of microglia in a cellular model of glioma-induced microglia activation, which simulates the interactions between brain tumor cells and microglia in the tumor microenvironment, the authors explored the impact of glioma-conditioned media (GCM) on microglia. When microglia are influenced by the tumor, they adopt a specific phenotype without inflammatory components, a process called “education” of microglia [72]. Accordingly, the group examined the effects of GCM on microglia in the presence of either **AD** alone or its complexes with siRNA, even at concentrations twice as high as those used in functional assays. In particular, microglial responses to GCM remained unaffected, highlighting the low toxicity of **AD** as a siRNA carrier for primary microglial cells. This lack of pro-inflammatory priming allowed for a more reliable evaluation of target gene knockdown, in particular of inhibitor of DNA binding 1 (Id1), a transcription regulator that plays a role in proliferation and differentiation in various cell types and is suggested to act as a master switch in microglia activation induced by factors derived from glioma [73]. Thus, by utilizing the **AD**-mediated delivery of a specific anti-Id1 siRNA, they successfully downregulated the target Id1 mRNA and its protein product in microglia. This enabled the team to explore the functions of Id1 in GCM-stimulated microglia for the first time. Transcriptome analysis revealed that silencing Id1 in microglia affected the expression of genes related to cell cycle and proliferation, while also uncovering a regulatory program involved in the expression of inflammatory genes, which was suppressed by glioma-derived factors.

Following the work described above, the same team with the additional contribution of other colleagues published a nature protocol paper in which they outline a manufacturing strategy for dendrimer **AD** and a siRNA delivery protocol targeting immune cells [74]. The synthesis of the dendrimer involves a straightforward click coupling process followed by an amidation reaction. The siRNA delivery protocol entails the mixing of siRNA and dendrimer in a buffer solution that is then applied to primary immune cells, ensuring efficient and functional siRNA delivery. Significantly, this dendrimer-mediated siRNA delivery approach surpasses conventional electroporation technology, opening up new possibilities for functional and therapeutic investigations of the immune system. The entire procedure encompasses multiple steps, including dendrimer production, which takes approximately 10 days, preparation of primary immune cells, with a duration ranging from 3 to 10 days depending on the tissue source and cell type, administration of dendrimer-mediated siRNA, and subsequent functional testing, requiring an additional 3 to 6 days. Successful implementation of this protocol could significantly advance the current understanding of immune system functions and pave the way for exploring novel therapeutic opportunities in immune-related research.

In another study, during a bilateral French–Italian collaboration the authors introduced a novel ionizable supramolecular dendrimer nanovector [75]. This nanocarrier is formed through the self-assembly of a small amphiphilic dendrimer and possesses ionizable tertiary amine terminals, resulting in a slightly positive surface charge at physiological pH, thereby enabling its electrostatic interaction with siRNA, forming a stable complex. The resulting siRNA/dendrimer delivery system exhibits an optimal surface charge that is neither neutral for preventing aggregation nor excessively high for causing cytotoxicity. This optimal charge facilitates the cellular uptake of siRNA and its release from endosomes. By employing this dendrimer-mediated siRNA delivery approach, the researchers achieved significant suppression of the MYC and AKT2 oncogenes, leading to a strong antiproliferative effect in various human cancer cell lines (including colorectal cancer Panc-1 cells, liver cancer HepG2 and Hep3B cells, and colorectal cancer HT-29 cells) and patient-derived cancer organoids. Notably, this delivery system outperformed the widely used Lipofectamine 2000, which is considered the gold standard vector. As such, the ionizable supramolecular dendrimer has great potential as a suitable siRNA delivery vector for efficient gene silencing and therapeutic applications.

## 4. Dendrimer-Decorated Metal Nanoparticles

**Figure 8:**
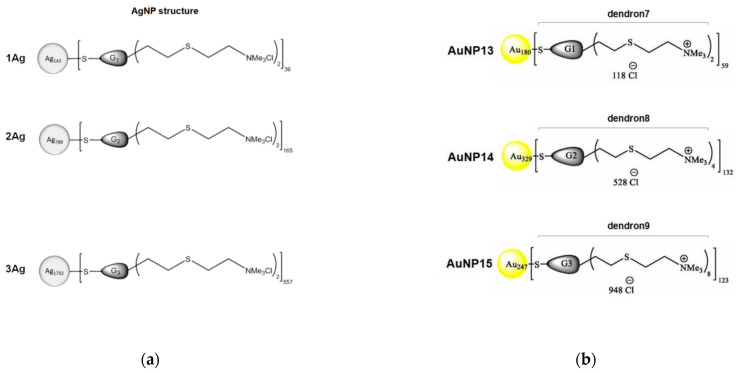
(**a**) Schematic structure of the Ag-based nanoparticles decorated with carbosilane dendrons. Adapted from [76], published by MDPI, 2020. (**b**) Schematic structure of the Au-based nanoparticles decorated with carbosilane dendrons. Adapted with permission from [77]. Copyright 2021, Elsevier.

A multinational team of scientists from Poland, Belarus, Germany, and Spain conducted a study proposing the use of carbosilane dendron-modified silver nanoparticles (AgNPs) (Figure 8a) as carriers to deliver anticancer siRNA targeting the antiapoptotic protein Bcl-xl (siBcl-xl) [76]. The researchers conducted several analyses to evaluate the complexes formed between AgNPs and siRNA. Gel electrophoresis (GE) was used to determine the molar ratios of dendron to siRNA that achieved maximum siRNA saturation by AgNPs. The ratios decreased with increasing nanoparticle/dendron generation, indicating complex formation through electrostatic interactions. Changes in the ZP during siRNA titration with AgNPs suggested that there were approximately one or fewer AgNPs per siRNA molecule, with nucleic acids wrapping around the positively charged NPs. TEM images supported the observation that the complexes had relatively large hydrodynamic diameters (around 500 nm), and the high polydispersity index indicated the formation of aggregates, particularly at higher dendron to siRNA molar ratios. The dendron-modified AgNPs exhibited concentration- and dendron-generation-dependent hemolysis and inhibition of lymphocyte proliferation. Hemolytic activity was prominent after 2 h of incubation at certain dendron concentrations, but complexation with siRNA reduced the level of hemolysis. The cytotoxicity of dendronized AgNPs in HeLa cells varied depending on the dendron generation, with the lowest toxicity observed for 1Ag. The cytotoxicity of 2Ag and 3Ag decreased after 24 h of incubation compared to the initial 3 h period, possibly due to the specificity of the LDH assay and cell growth. Although AgNP–siRNA complexes were able to deliver siRNA into cells, no increased cytotoxic activity was observed compared to that of free nanoparticles. The authors hypothesized that hindered endosomal escape, limited siRNA release from complexes, or insufficient intracellular delivery of siRNA could be responsible for this result. Additionally, the low expression levels of Bcl-xl and other Bcl-2 family proteins in HeLa cells posed challenges in assessing the effectiveness of the siRNA delivery system. Therefore, further research is needed to optimize the delivery system, select appropriate therapeutic nucleic acids for specific cellular models, and overcome these limitations.

In a related study, the same research group investigated gold nanoparticles (AuNPs) of different sizes coated again with cationic carbosilane dendrons (Figure 8b) as vehicles for siRNA delivery [77]. The formation of complexes between AuNPs and siRNA was thoroughly characterized using various techniques. Stable complexes were formed, and the specific molar ratios depended on dendron generation. Heparin was able to release siRNA from the complexes, but middle-sized AuNPs showed only partial release, suggesting a strong electrostatic interaction among dendrons, AuNPs, and heparin. Complexation with dendrons and AuNPs protected siRNA against degradation by RNase. The size of the complexes varied depending on the analysis method, and larger complexes were formed with certain AuNPs because of differences in the core and coating sizes of the nanoparticles. The presence of AuNPs and siRNA complexes inside cells was confirmed by ultrastructure analysis and confocal microscopy, with endocytosis identified as the primary uptake pathway. The cytotoxicity assessment on four cell lines demonstrated that cytotoxicity depended on the dendron generation rather than the metallic core. Higher generation dendrons exhibited increased cytotoxicity, with pure dendrons being more toxic than dendronized AuNPs. This observation was attributed to the redistribution of cationic charges upon binding to AuNPs. The effectiveness of AuNP–siRNA complexes in cell lines was investigated using the intermediate-size AuNPs and two siRNAs targeting Bcl-xl and Mcl-1. SiMcl-1 had a significant impact on three cancer cell lines, while siBcl-xl showed limited effectiveness, likely due to the variation in overexpression and activity levels of the targeted proteins in different cancer cell lines. In conclusion, the authors suggested that modifications such as PEGylation may be necessary to improve the biocompatibility and targeted delivery of nucleic acids.

In a follow-up article, the same joint research team further studied the carbosilane dendron-decorated AuNPs shown in Figure 8b to better evaluate their efficacy in delivering siRNAs to tumor cells using a photometric viability test and FC [78]. They assessed both the effectiveness of the complexes in inducing apoptosis and the overall impact of proapoptotic siRNAs on cells. Among the tested AuNPs, the intermediate-size NPs again exhibited the highest effectiveness and toxicity. The delivery efficiency of siRNAs to suspension cell lines ranged between 50% and 60%. Compared to the control group, complexes containing targeted siRNAs led to a 20% decrease in cell viability and initiated apoptosis. In order to increase the siRNA delivery potential and efficacy in gene silencing of these NPs, the authors finally proposed that further NP modifications with peptides, oligosaccharides, or other components that facilitate the delivery and release of nucleic acids could ultimately lead to better biological performance.

## 5. Self-Assembling Dendrimers in Cancer Bioimaging

**Figure 9:**
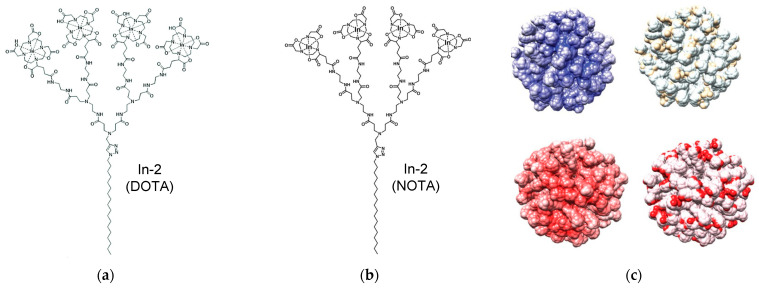
(**a**) Chemical structure of the self-assembling amphiphilic dendrimers **In-2(DOTA)** bearing radionuclide In^3+^ terminals complexes with the macrocyclic DOTA cage. Adapted with permission from [81]. Copyright 2019, John Wiley & Sons. (**b**) Chemical structure of the self-assembling amphiphilic dendrimers **In-2(NOTA**) bearing radionuclide In^3+^ terminals complexes with the macrocyclic NOTA cage. Adapted with permission from [82]. Copyright 2020, The Royal Chemical Society, 2020. (**c**) Top row: electrostatic surface potential (**left**) and representation of the surface charge distribution localized on the In^3+^/NOTA complexes at the NOTA terminals (neutral, ivory) (**right**) for the **In-2(NOTA)** dendrimer nanomicelles. Bottom row: electrostatic surface potential (**left**) and representation of the surface charge distribution localized on the In^3+^/DOTA complexes at the DOTA terminals (neutral, ivory) (**right**) for the **In-2(DOTA)** dendrimer nanomicelles. In the top-left and bottom-left panels, the red color represents a negatively charged surface, the dark blue color represents a positively charged surface, while the white color represents a neutral surface, respectively. Adapted with permission from [82]. Copyright 2020, The Royal Chemical Society.

The field of bioimaging has revolutionized medicine by providing accurate diagnostic and treatment information for human pathologies. Researchers are actively developing new biomarkers for functional and molecular imaging to improve the assessment of targeted therapy. Molecular imaging combines techniques from biomedical imaging and molecular biology to noninvasively visualize and quantify the spatial and temporal distribution of biological processes in living organisms. This approach has applications in biochemistry, biology, diagnostics, and therapeutics. Examples of molecular imaging techniques include molecular magnetic resonance imaging (mMRI) and spectroscopy, positron emission tomography (PET), single-photon emission computed tomography (SPECT), optical imaging (such as optical bioluminescence and optical fluorescence), photoacoustic imaging, and multimodal imaging, among others. Some modalities, like radionuclide and optical imaging, require the administration of molecular probes to generate the imaging signal. On the other hand, mMRI and photoacoustic imaging can track the effectiveness of drugs using naturally occurring molecules or externally administered molecular probes [79,80]. In this context, a French and Italian team has developed supramolecular nanosystems for SPECT bioimaging by designing self-assembled amphiphilic dendrimers optimized to incorporate multiple In^3+^ radionuclides, which serve as SPECT reporting agents, at their terminal positions [81,82]. In a first effort, the group produced an amphiphilic molecule with the cyclic chelator DOTA (1,4,7,10-tetraaza-cyclododecane-1,4,7,10-tetraacetic acid) on its surface (In-2(DOTA), Figure 9a) [81]. The stoichiometric binding of four In^3+^ ions per molecule of dendrimer 2(DOTA) was verified using high-resolution mass spectrometry (HRMS) and ITC, the latter technique also providing detailed information on the binding thermodynamics between the metallic cations and the host dendrimers. The spontaneous self-assembly of **In-2(DOTA)** in solution was then investigated, revealing the formation of spherical negatively charged nanomicelles (~18 nm in diameter) as observed by TEM and DLS. MD simulations provided additional support for the spontaneous aggregation of **In-2(DOTA)** into spherical micelles, the terminal groups of In^3+^/DOTA being consistently located at the periphery of the micelles without backfolding. Finally, the radioactive dendrimer complex **[^111^In]In-2(DOTA)** was prepared, with a radiochemical purity of over 91 ± 2% and a high molar activity of 1.09 ± 0.15 GBq mmol that remained stable for up to 30 h at 37 °C in human serum. Taking advantage of these properties, SPECT imaging using **[^111^In]In-2(DOTA)** was successfully performed in mice with orthotopically xenografted tumors derived from a human pancreatic adenocarcinoma cell line (SOJ-6). SPECT signals were precisely coregistered with computerized tomography (CT) for accurate anatomical localization, facilitating further quantification.

However, the authors reported a limitation of **[^111^In] In-2 (DOTA)** in terms of its relatively high accumulation in the liver and kidneys, as this finding could potentially restrict its clinical translation in the future. Since the biodistribution of radiotracers can be significantly influenced by different chelators, which are determined by factors such as size, charge, geometry, and lipophilicity when combined with metal ions, the researchers speculate that, in the case of the DOTA chelator, the negatively charged complex formed with In^3+^ may contribute to the observed high uptake and retention of the In-2 nanomicelle in the liver, despite its small size. Accordingly, they hypothesized that reducing the negative surface charge of the dendrimer could potentially mitigate this undesired liver retention. Therefore, by replacing the larger macrocyclic DOTA cage with the smaller 1,4,7-triazacyclononane-1,4,7-triacetic acid (NOTA) scaffold as the chelator for In^3+^, the resulting dendrimer **In-2(NOTA)** (Figure 9b) featured neutral In^3+^ complexes at the terminals [82]. The same experimental protocol was employed to investigate the thermodynamics of the interaction between In^3+^ and dendrimer 2(NOTA), which confirmed also in this case the formation of small spherical nanomicelles with dimensions and In^3+^ binding stoichiometry/thermodynamics very similar to those reported for the DOTA-terminated dendrimer. However, as mentioned, this change in surface charge transformed the ZP of the nanosystems from negative (**In-2(DOTA)**) to positive (**(In-2(NOTA)**) (Figure 9c). Surprisingly, this alteration in surface charge has significant implications for the biodistribution profile of the SPECT nanoprobe. Contrary to the prevailing scientific consensus that positively charged nanoparticles tend to accumulate in the liver, the modified dendrimer-based nanosystems exhibit an entirely different pattern. They demonstrate highly desirable biodistribution with significantly reduced accumulation in the liver, leading to greatly improved tumor imaging capabilities. SPECT imaging was then performed again in mice with orthotopically xenografted SOJ-6 tumors using **[^111^In]In-2(NOTA)**, while **[^111^In]In-2(DOTA)** was used as a control, and the results clearly indicated a marked improvement in image contrast and enhanced visualization of tumors with **[^111^In]In-2(NOTA)** compared to its DOTA counterpart. The enhanced tumor imaging achieved with **[^111^In]In-2(NOTA)** was further validated using a patient-derived pancreatic cancer xenograft model (L-IPC cell line). Quantification of the μSPECT signals for **[^111^In]In-2(NOTA)** in the liver demonstrated a significant reduction (more than halved) compared to **[^111^In]In-2(DOTA)** as early as 2 h after injection, and this reduction was maintained at 24 and 48 h post-injection. Notably, **[^111^In]In-2(NOTA)** significantly reduced µSPECT signals in organs that typically contribute to background noise, such as the heart, lungs, brain, muscle, and bladder, at 24 and 48 h post-administration compared to 2 h post-injection, resulting in improved tumor imaging quality. Importantly, the administration of radioactive **[^111^In]In-2(NOTA)** to mice during the experimental imaging procedures did not result in any abnormal behaviors or adverse effects. Similarly, non-radioactive **In-2(NOTA)** administered at a dosage ten times higher than required for SPECT imaging did not cause organ damage or abnormalities in blood biochemistry. Histological examination of organs from **In-2(NOTA)** treated mice revealed no observable lesions or significant pathological changes. Furthermore, the levels of various blood biochemical parameters remained comparable to those of untreated mice, indicating normal liver, kidney, and muscle function. These results confirm the well-tolerated nature of **In-2(NOTA)** and its ability to deliver effective SPECT imaging quality without inducing adverse effects.

## 6. Covalent Dendrimers, Self-Assembling Dendrimers, and Dendrimer Decorated Metal Nanoparticles for Applications beyond Cancer

### 6.1. Covalent and Self-Assembling Dendrimers with Antibacterial Activity

**Figure 10:**
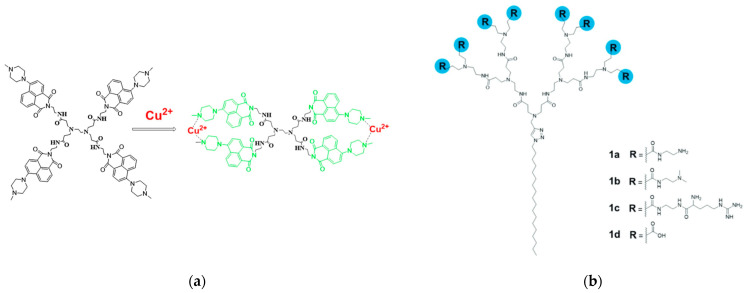
(**a**) Chemical structure of the metallodendrimer [Cu_2_(D)(NO_3_)_2_]. Adapted with permission from [85]. Copyright 2021, Elsevier. (**b**) Chemical structures of the self-assembling amphiphilic dendrimers **1a–d**. Adapted with permission from [86]. Copyright 2022, The Royal Society of Chemistry.

The issue of antibiotic resistance poses a significant worldwide health concern as it involves the transmission of bacteria and genetic elements among humans, animals, and the environment. Despite the presence of various barriers, the transfer of both bacteria and genes continues to occur, leading to the acquisition of new resistance traits by pathogens. This constant evolution of antibiotic resistance hampers our ability to effectively prevent and treat bacterial infections [83,84]. Therefore, it is of utmost importance to gain insight into the molecular mechanisms employed by bacteria to evade the effects of antimicrobial agents. This understanding will allow the identification of global trends in resistance and will enhance the utilization of existing drugs. It also aids in the development of new drugs that are less prone to resistance development and the formulation of innovative strategies to effectively address antibiotic resistance. In this field, a fluorescent dendrimer was synthesized by modifying a zero-generation PAMAM dendrimer at the periphery during a collaboration between Italian and Bulgarian scientists [85]. The modifications involved substituting the C-4 position with 1,8-naphthalimide units that contained N-methylpiperazine, followed by the addition of Cu(II) ions to create the metallodendrimer [Cu_2_(D)(NO_3_)_2_] (Figure 10a). Various techniques such as UV-vis, fluorescence spectroscopy, FT-IR, NMR, and SEM were utilized to determine the precise structure of the dendrimer ligand and the metallodendrimer. The photophysical properties of the resulting yellow-green fluorescent dendrimer were examined in organic solvents with different polarities, revealing a photoinduced electron transfer (PET) process that was particularly pronounced in polar solvents. Furthermore, fluorescence studies conducted in an ethanol–water solution at different pH levels showed a fluorescence intensity of the dendrimer that is more than seventeen times higher in an acidic medium. To assess the dendrimer’s ability to bind to Cu(II), EPR studies were conducted by varying the [Cu(II)/dendrimer external sites] molar ratio in a DMSO solution. The EPR results indicated the formation of a square-planar Cu-N_2_O_2_ coordination at the interface of the internal and external regions of the dendrimer, involving nitrogen sites such as N-CH_3_ groups, amine groups, and amide groups, along with the presence of two solvent molecules. Copper (II) ions were distributed between the dendrimer interface and the external surface, with saturation of the nitrogen sites at the interface occurring in a ratio of 1:2 M between Cu(II) and methylpiperazine units. The antimicrobial activity of both the dendrimer ligand and its Cu(II) complex was evaluated against various strains of bacteria and yeast using agar-based assays, liquid medium tests, and deposition onto cotton fabric. The results demonstrated the effective antibacterial properties of the dendrimer ligand, which were further enhanced by its complexation with Cu(II).

In the quest of finding new dendrimer-based antibacterial agents, a group of colleagues for France, Italy and Israel decided to challenge an amphiphilic dendrimer with different terminal functionalities (Figure 10b) for activity against both Gram+ and Gram- bacteria [86]. Dendrimers **1a–d** self-assembled in water, forming small, spherical nanomicelles with dimensions in the range from 10 to 20 nm (as confirmed by TEM), and ZP values ranging from +35 mV to −13 mV, indicating differences in their chemical composition. The cytotoxicity of all dendrimers was evaluated in fibroblast (L929) and kidney (HEK293) cell lines, revealing that dendrimers **1a**, **1b**, and **1d** had no significant adverse effects at concentrations of up to 200 μM. However, dendrimer **1c** exhibited notable toxicity towards both cell types. Similarly, the hemolytic activity of the dendrimers on red blood cells was examined, showing that dendrimer **1c** had the highest hemolytic activity with an IC_50_ value of 50 μM, while dendrimers **1a**, **1b**, and **1d** had lower hemolysis, with IC_50_ values of 100 μM for **1a** and **1b**, and no notable hemolysis for **1d**. The observed toxicity of dendrimer **1c** was attributed to the arginine terminals, which interact strongly with the cell membrane through electrostatic interactions and bivalent hydrogen bonds. Among the 4-dendrimer series, the amphiphilic dendrimer containing amine terminals (**1a**) showed remarkable activity against the two distinct types of bacteria as well as drug-resistant bacteria and inhibited the development of biofilms. The strong antibacterial activity of dendrimer **1a** could be attributed to its self-assembling properties, as the hydrophilic dendrimer counterpart lacking the alkyl chain showed no antibacterial activity. The dendrimer interacts and binds via electrostatic interactions with the bacterial membrane, where they dynamically self-assemble into supramolecular nanoaggregates for stronger and multivalent interactions, as demonstrated by multidisciplinary studies combining experimental approaches and computer modeling. In particular, molecular simulations of **1a** were able to explain why this amphiphilic molecule was already active against the selected bacteria strains at a minimum inhibitory concentration (MIC) value (3.1 μM) lower than its critical micellar concentration (CMC) value (15 μM). These MD simulations indeed showed that, starting from an ensemble of **1a** randomly distributed in a water shell above the membrane (mimicking a situation below the CMC), the amphiphiles approached the negatively charged bacterial membrane surface where they accumulated due to mutual electrostatic interaction. The MD run ended with several clusters of **1a** aggregates on the bacterial surface, which protected their hydrophobic tails from water and allowed positively charged dendrons to bind strongly to bacterial membranes via cooperative and multivalent electrostatic interactions. A second round of atomistic MD simulations on these aggregates showed **1a** disassembling and spreading across the upper membrane leaflet, ultimately promoting the full insertion of their hydrophobic tails into the bilayer of the bacterial membrane. These in silico experiments support the mechanistic idea of **1a** molecules binding to and becoming enriched on the bacteria membrane via favorable electrostatic/polar interactions before self-assembling into nanoclusters due to their amphiphilicity. These clusters eventually rearrange, distributing dendrimer molecules around the bacterial surface to allow the long hydrophobic tails to fully integrate into the lipid bilayer via collective hydrophobic interactions, creating robust antibacterial activity. The dynamic self-assembling feature of dendrimer **1a**, which forms nanomicelles on the surface of the bacterial membrane, thereby inducing bacterial cell lysis and, hence, antibacterial action, has potential for nanotechnology-based antibiotic delivery and gives a fresh perspective for combating resistant bacterial illness.

### 6.2. Dendrimer-Decorated Metal Nanoparticles with Antiviral Activity

In a collaborative study involving researchers from Slovakia, Poland and Spain, Gaiarova et al. investigated the interaction between gp160 synthetic peptides derived from the human immunodeficiency virus (HIV) envelope and three different dendronized AuNPs labeled as **AuNP13**–**AuNP15** (as shown in Figure 8b) [87]. The results of the study revealed that the HIV peptides exhibited interactions with the AuNPs, leading to changes in their secondary structures. Several observations supported this interaction: the attached fluorescent dye showed restricted mobility, fluorescence polarization measurements indicated an increase in peptide helicity, and CD analysis confirmed alterations in peptide conformation. TEM imaging displayed the formation of complex structures resembling cloud-like formations with interconnected nanoparticles. The binding of the negatively charged peptides to dendronized AuNPs was dependent on the number of functional groups present. Additionally, DLS, laser Doppler velocimetry (LDV), and agarose GE experiments indicated that the nanoparticle with the highest degree of dendronization exhibited the fastest binding to negatively charged peptides and hindered their migration. Overall, these findings emphasize the potential of dendronized gold nanoparticles as a promising platform for HIV peptide-based immunization, offering a viable approach for the development of effective HIV vaccines.

### 6.3. Impact of Covalent Dendrimers and Dendrimer-Decorated Metal Nanoparticles on the Immune System

In a Polish–German–Spanish study [88], the researchers aimed to investigate the induction of pyroptosis, an inflammatory form of programmed cell death, in immune cells, particularly macrophages, by combining dendronized AuNPs with bacterial lipopolysaccharides (LPSs) known for their pro-inflammatory properties. The results demonstrated that the decorated AuNPs promoted caspase-1 activity (3–4 times higher than the control) and enhanced the release of interleukin (IL)-18 and IL-1β, which are pro-inflammatory cytokines. Importantly, the dendronized AuNPs did not cause gasdermin D cleavage or subsequent pore formation. Notably, the production of pro-inflammatory cytokines was predominantly observed during LPS treatment, while their secretion occurred only after the administration of dendronized AuNPs (up to 80 pg/mL of IL-1β released into the supernatant). These findings indicate that dendronized AuNPs can induce inflammatory mechanisms resembling pyroptosis, and the presence of bacterial LPS potentiates these mechanisms. Furthermore, the intensity of this effect was found to depend on the surface modification of the AuNPs. These results provide valuable insights into the cytotoxicity of metal nanoparticles, including their impact on immune responses, highlighting the significant role of surface modifications in their nanotoxicological effects.

The advancement of personalized medicine and new treatment approaches requires the development of innovative pharmacological tools, such as nanoparticles that can directly modulate the immune system [89,90]. In the framework of a Spanish, German, and Polish collaboration, Jatczak-Pawlik and colleagues created a unique type of nanoparticles called mannose-functionalized poly(propyleneimine) glycodendrimers [91]. These nanoparticles were designed with mannose molecules attached to poly(ethylene glycol)-based linkers, which were then coated with maltose. This novel design significantly increased the effectiveness of the glycodendrimers and had various effects on myeloid cells. Specifically, the mannose glycodendrimers stimulated the production of interleukin-8 (IL-8) in HL-60 and THP-1 cells. In HL-60 cells, the induction of IL-8 was primarily caused by activating specific genes through binding of the activator protein 1 (AP-1) transcription factor to the promoter region. On the other hand, in THP-1 cells, which originally had lower levels of IL-8, induction was achieved mainly by stabilizing mRNA molecules. The successful achievement of targeted immunomodulation, based on the idea that mannose-modified dendrimers can act as external regulators of pro-inflammatory chemokine levels, may open up new opportunities for the development of bioactive nanoparticles with innovative functionalities.

### 6.4. Covalent and Self-Assembling Dendrimers in Binding Other Biological Molecules

**Figure 11:**
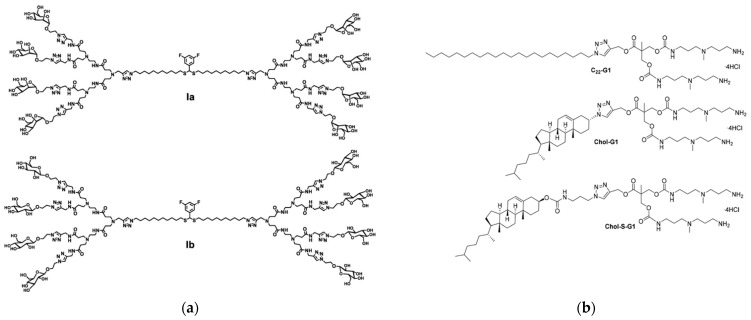
(**a**) Chemical structure of the bola-amphiphilic glycodendrimers with mannose (**Ia**) and glucose (**Ib**) terminals. Adapted with permission from [92]. Copyright 2022, John Wiley & Sons. (**b**) Chemical structures of the self-assembling multivalent (SAMul) amphiphilic dendrons. Adapted with permission from [93]. Copyright 2019, The Royal Society of Chemistry.

Dendrimers have great potential to create carbohydrate mimics with distinct multivalent cooperation. Thus, researchers from France, Italy, Poland, China, and Taiwan made a collaborative effort to modify the surface groups of the dendrimer **bola8a** (depicted in Figure 5d) with mannose (**Ia**) and glucose (**Ib**) terminals (Figure 11a) [92]. These dendrimers were easily synthesized by sequential click reactions and exhibited favorable properties such as water solubility and biocompatibility. The unique structure of **Ia** resulted in a high affinity for the carbohydrate binding protein concavalin A (ConA). Furthermore, **Ia** specifically targeted the mannose receptor, while **Ib** selectively targeted the glucose transporter. Both **Ia** and **Ib** demonstrated effective uptake by microglia, astrocytes, and cancer cells. Overall, these bola-amphiphilic glycodendrimers show great potential for targeted drug delivery to cells expressing carbohydrate-binding proteins.

The formation of micelles through the self-assembly of cationic amphiphilic dendrons is highly effective in binding important polyanions such as heparin. In medical applications such as coagulation control, where protamine is currently used, a synthetic heparin rescue agent could be beneficial. However, micelles can have limited stability in serum and can exhibit undesirable toxicity profiles. In their research, Tena-Solsona and colleagues from the United Kingdom, Italy, and Spain described the optimization of self-assembled multivalent (SAMul) amphiphilic dendron arrays (Figure 11b) to enhance heparin binding under competitive conditions [93]. They specifically focused on modifying the hydrophobic portion of the amphiphile to kinetically stabilize the self-assembled nanostructures, preventing significant loss of binding capacity in the presence of human blood. Of particular importance, the inclusion of cholesterol hydrophobic units outperformed the systems utilizing a simple aliphatic chain. Additionally, the presence of the aliphatic chain altered the binding thermodynamics with HSA. Molecular simulations further supported these findings, revealing that aliphatic lipids can be more easily extracted from the relevant self-assembled nanostructures compared to cholesteryl ester equivalents. This aligns with the experimental observation that cholesterol-based systems disassemble and degrade at a slower rate via ester hydrolysis. Moreover, the stabilization of SAMul nanostructures not only reduced the toxicity toward human cells but also improved biocompatibility, resulting in a significant improvement in the survival of human hepatoblastoma cells. These findings highlight the advantages of modifying the hydrophobic portion of amphiphilic dendrons, specifically with cholesterol units, to improve stability, biocompatibility, and binding capabilities in the presence of physiologically relevant substances like heparin.

### 6.5. Covalent Dendrimers againsts Systhemic Lupus Erythematosus

Antibodies directed against double-stranded (ds) DNA are used as a serological marker for systemic lupus erythematosus (SLE), a condition in which these antibodies bind to (ds) DNA in the bloodstream. This leads to the formation of immunocomplexes that spread throughout the body, contributing to lupus glomerulonephritis and other symptoms. To address the pathological manifestations associated with SLE, it is crucial to disrupt or inhibit the formation of these immunocomplexes. In a German, Italian and American effort, different generations of maltose-decorated PAMAM and PPI dendrimers, along with two oligopeptides containing polyethylene glycol units, were synthesized, characterized, and evaluated for their anti-SLE activity [94]. The activity of glycodendrimers and oligopeptides was evaluated using enzyme-linked immunosorbent assay (ELISA) and EPR techniques in human plasma samples from SLE patients and healthy individuals. Various methods for immunocomplex formation were examined. The results demonstrated that both types of glycodendrimers and oligopeptides effectively inhibited the formation of immunocomplexes, and some disassembly of preformed immunocomplexes was observed. Glycodendrimers exhibited superior activity compared to oligopeptides, with the third generation PPI dendrimer showing the most promising potential for anti-SLE therapy. This study highlights the potential for the development of a novel class of dendritic therapeutics for preclinical investigations aimed at treating SLE.

## 7. Conclusions

COST Action CA17140 was a pioneering and interdisciplinary network that brought together individuals from academic institutions, small and medium enterprises (SMEs) and clinical research organizations (CROs). Its main focus was on the entire process of developing nanosystems for the delivery of anticancer drugs, starting from their initial design and progressing to preclinical testing for effectiveness, pharmacokinetics, and toxicity. Additionally, the network aimed to create detailed protocols required for the initial phase of clinical studies. The CA17140 network achieved significant success by establishing core research teams that produced high quality work and made important contributions to funding applications and scientific discoveries. They actively promoted the potential of nanomedicine through various means, such as publishing numerous articles in reputable international peer-reviewed journals and participating in open events. Their efforts resulted in the publication of more than 100 articles, many of which appeared in top-tier journals. CA17140 was committed to providing exceptional training opportunities for young European investigators and innovators, without any form of discrimination based on nationality, age, or gender. The network put a strong emphasis on training the next generation of researchers. Notably, a large portion of the grants for Short-Term Scientific Missions (STSMs, 88%) and Training Schools (100%) were awarded to PhD students and young researchers. These opportunities were accessible to individuals from less research-intensive countries (ITCs), and the gender distribution among the trainees was relatively equal, with 62% being female students. The network played a crucial role in supporting the career advancement of Ph.D. students and young researchers by providing various strategies, including appointing individuals to leadership positions and involving them in organizations, scientific committees, and action conferences. This inclusive approach resulted in job offers and promising career prospects for these young scientists. Throughout the duration of CA17140, numerous job opportunities emerged at different levels, allowing individuals with Ph.D. and young researchers to pursue careers in science and research at prestigious EU institutions, including academic institutions and SMEs. SME representatives made significant contributions to CA17140. They held leadership roles, acted as hosts and recipients of STSM grants, and collaborated on research grant proposals, some of which received funding. Industrial partners were also featured in several publications resulting from collaborations within CA17140. Finally, CA17140 actively engaged with the European Parliament and the Directorate General of Health of the European Commission regarding the inclusion of nanomedicines and nanosimilars in the current proposal for the Regulation of the European Parliament. This proposal is of great societal and policy importance as it pertains to the EU’s processes for authorizing and monitoring medicinal products for human use, as well as establishing guidelines for the European Medicines Agency. The proposed legislation includes revisions to existing regulations and the repeal of certain previous regulations related to medicinal products.

This review aimed to offer only a glimpse of the intense work performed by the CA17140 network in the vast field of nanomedicine, with a specific focus on dendrimers and dendrons as nanocarriers and/or nanomedicines per se. These fascinating, highly branched macromolecules have emerged relatively recently, but hold great promise as nanocarriers for drug delivery, theranostic agents, and gene vectors, both in pharmaceutical research and innovation and in other healthcare applications. Despite significant progress in dendrimer chemistry and applications since their discovery, the synthesis, development, and design of pure and safe dendrimer-based products still pose significant challenges in the field. With the wealth of knowledge produced during the four and a half years of activity within our COST Action, we hope that the CA17140 community has successfully contributed to filling some of the existing gaps and provided some new tools to advance this science along the hard road of clinical translation of dendrimer-based nanomedicines.

## Data Availability

Not applicable.
